# Supplementary material for: Hedgehog artificial macrophage with atomic-catalytic centers to combat Drug-resistant bacteria
Source: Nat Commun. 2021 Oct 22;12:6143. doi: 10.1038/s41467-021-26456-9 (PMC8536674; doi:10.1038/s41467-021-26456-9)
Supplement: Supplementary file 1 — Supplementary Information [file 41467_2021_26456_MOESM1_ESM.pdf]

Supplementary Information for

Hedgehog Artificial Macrophage with Atomic-Catalytic Centers to Combat Drug-Resistant Bacteria

Yanping Long,[1] *Ling Li,*[2, 3] *Tao Xu,*[1] *Xizheng Wu,*[1] *Yun Gao,*[4] *Jianbo Huang,*[2] *Chao He,*[1] *Tian Ma,*[2] *Lang Ma,*[2] *Chong Cheng,*[1,]* *& Changsheng Zhao*[1,5,6,]*

[1] College of Polymer Science and Engineering, State Key Laboratory of Polymer Materials Engineering, Sichuan University, Chengdu, 610065, China

[2] Department of Ultrasound, West China Hospital, Sichuan University, Chengdu, 610041, China

[3] Department of Ultrasound, Affiliated Hospital of North Sichuan Medical College, Nanchong, 637000, China

[4] College of Biomass Science and Engineering, Textile Institute, Sichuan University, Chengdu, 610065, China

[5] College of Biomedical Engineering, National Engineering Research Center for Biomaterials, Sichuan University, Chengdu, 610064, China

[6] College of Chemical Engineering, Sichuan University, Chengdu, 610065, China

* Corresponding author. Tel: +86-28-85400453, Fax: +86-28-85405402,

E-mail: (C. Cheng) chong.cheng@scu.edu.cn; (C.S. Zhao) zhaochsh70@scu.edu.cn

Supplementary Figures

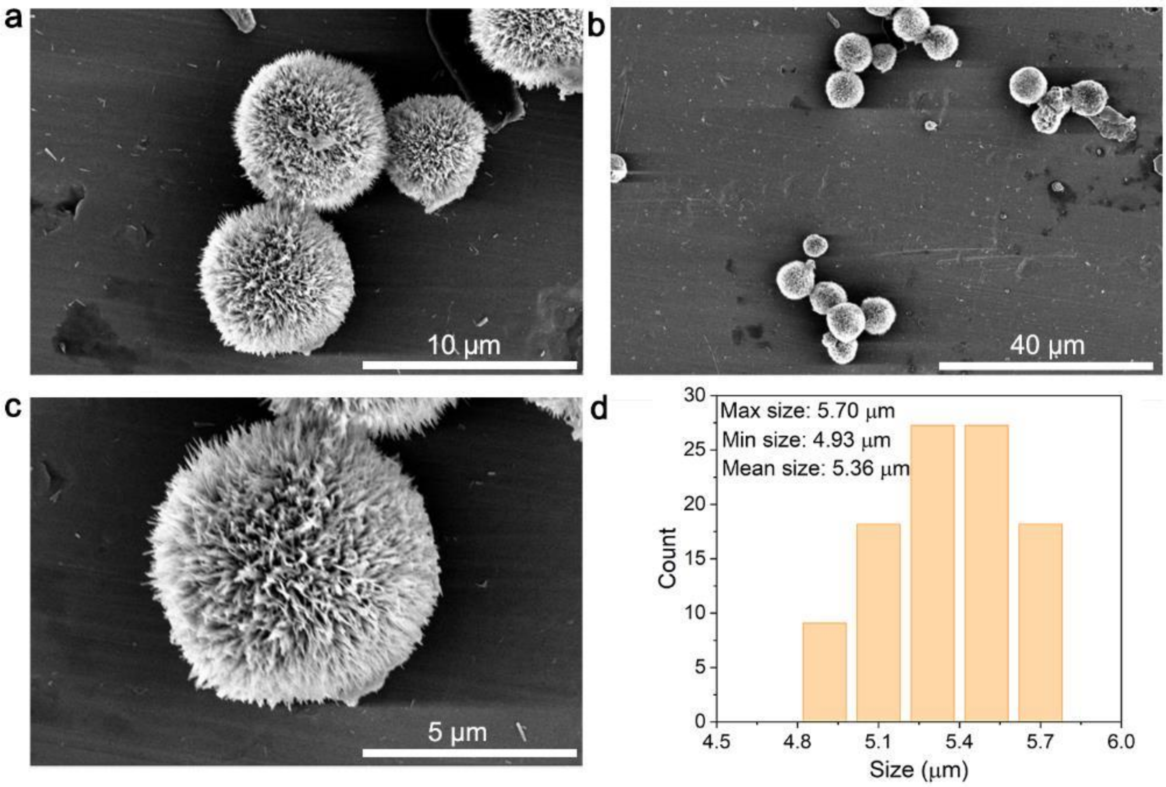

Supplementary Figure 1. **a-c** The SEM images and **d** corresponding average sizes of Art M before carbonization. Experiments were repeated independently **a**, **b**, **c** three times with similar results. The amount of urea (modifier) is 25 mg.

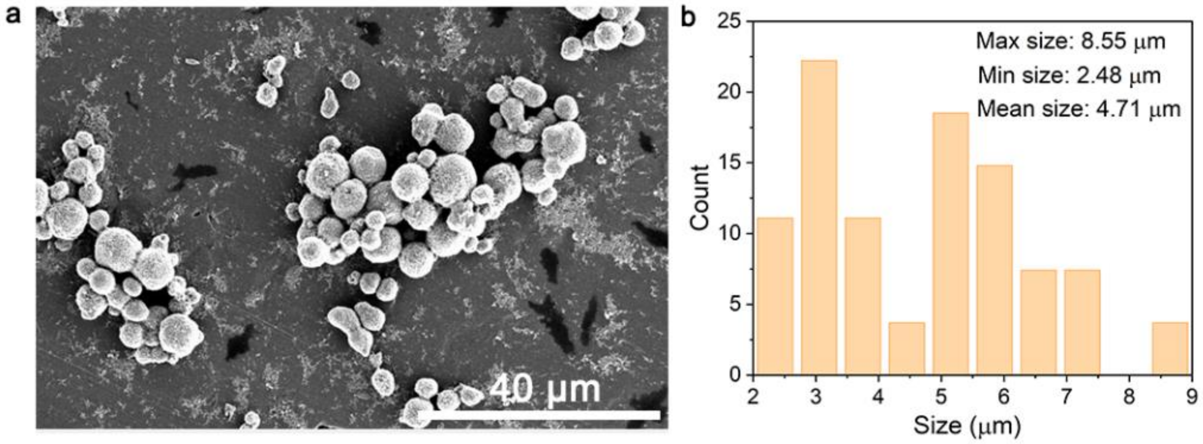

Supplementary Figure 2. **a** The SEM images and **b** corresponding average sizes of Art M before carbonization. Experiments were repeated independently **a** three times with similar results. The amount of urea (modifier) is 100 mg.

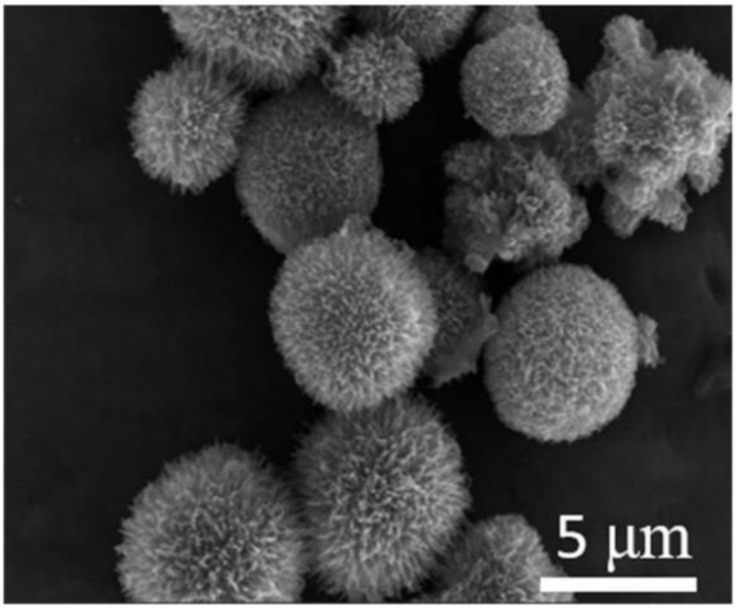

Supplementary Figure 3. The SEM of Art M after carbonization. Experiments were repeated independently three times with similar results. The amount of urea (modifier) is 25 mg.

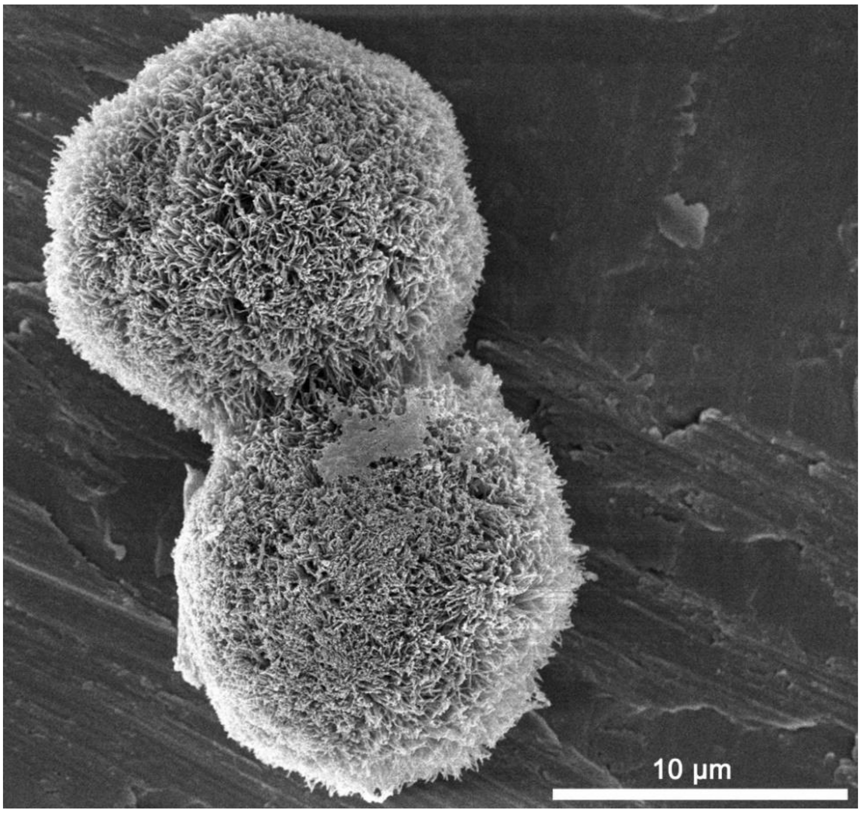

Supplementary Figure 4. The SEM image of V-Art M. Experiments were repeated independently three times with similar results.

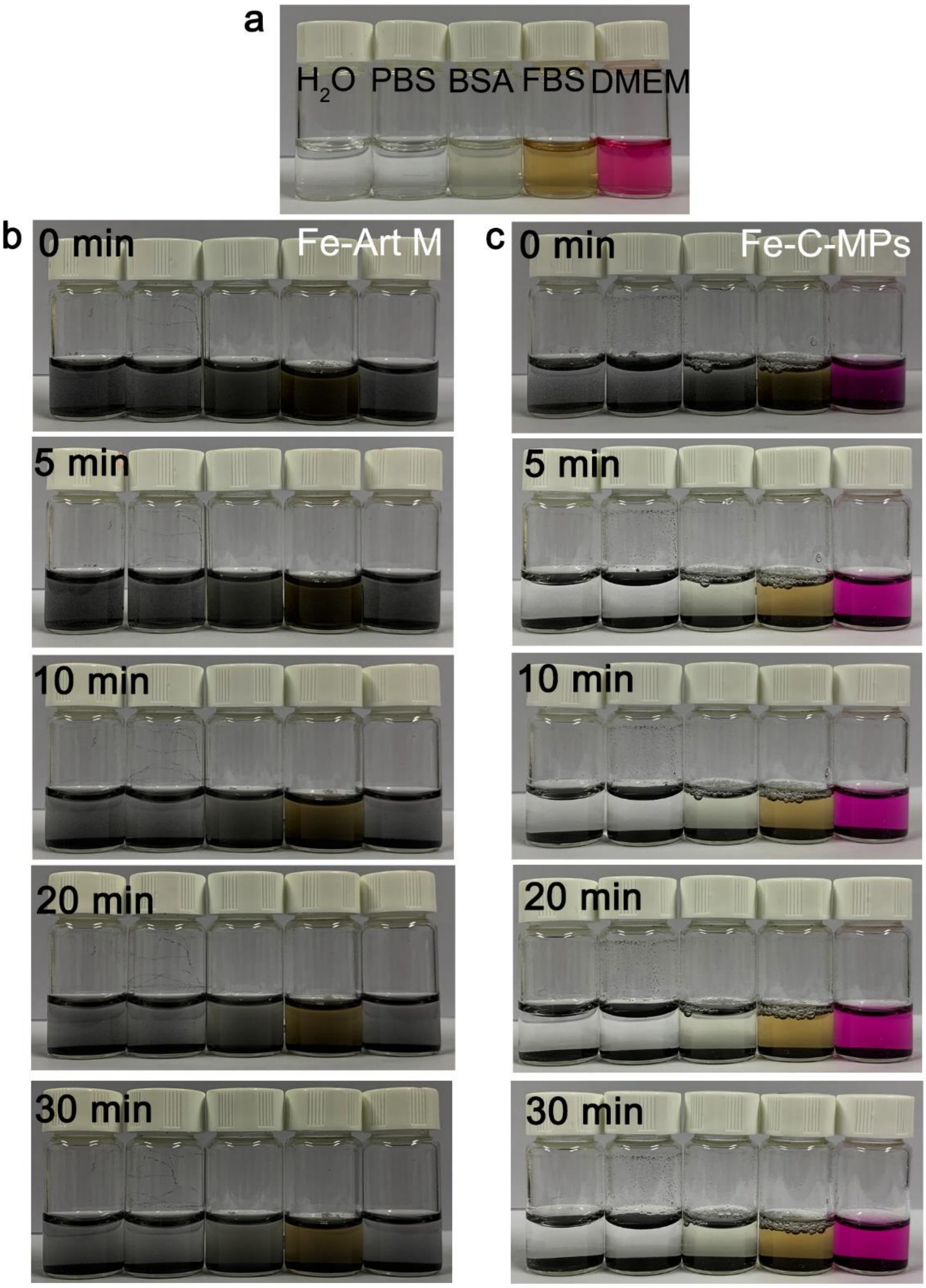

Supplementary Figure 5. Digital photographs of **a** original solutions, **b** Fe-Art M suspensions, and **c** Fe-C-MPs suspensions for varied durations (including 0, 5, 10, 20, and 30 min). After 5 min, the micrometer-scale Fe-C-MPs all settled, while Fe-Art M can maintain a suspension for about 20-30 min, thus showing that the dispersibility of Fe-Art M (hedgehog topography) is better than that of Fe-C-MPs (smooth surface). Experiments were repeated independently per group three times with similar results. PBS: phosphate buffer saline; BSA: bovine serum albumin; FBS: fetal bovine serum; DMEM: Dulbecco's modified eagle medium. It is noticed that after adding Fe-Art M, the DMEM solution becomes transparent due to its porous structure induced high adsorption capability.

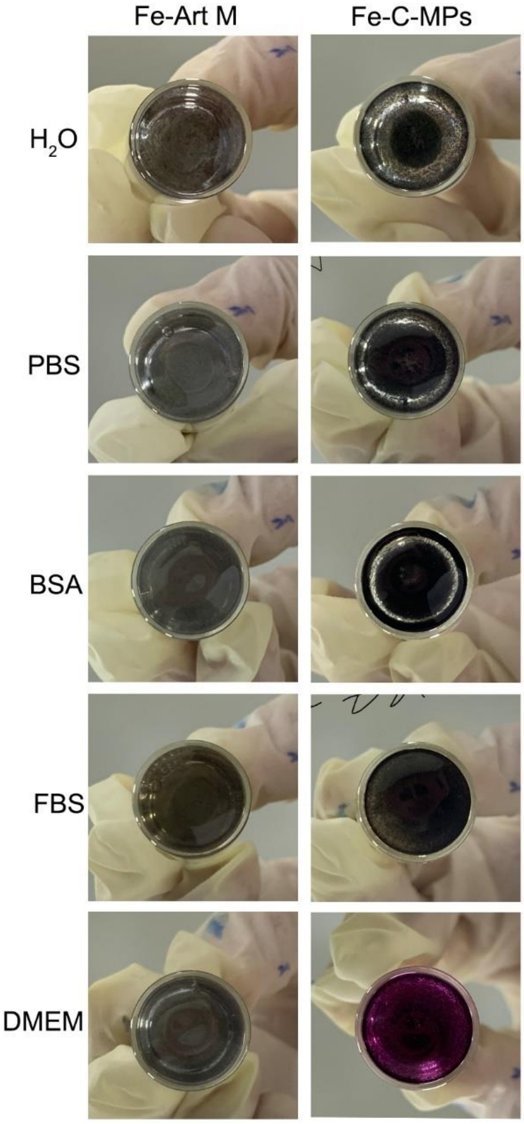

Supplementary Figure 6. The bottom of Fe-Art M and Fe-C-MPs solutions. The duration time is 5 min. Compared to a large amount of sediment in the Fe-C-MPs (smooth surface) group, the Fe-Art M shows almost no precipitant, thus indicating the enhanced dispersibility due to hedgehog topography. Experiments were repeated independently three times with similar results.

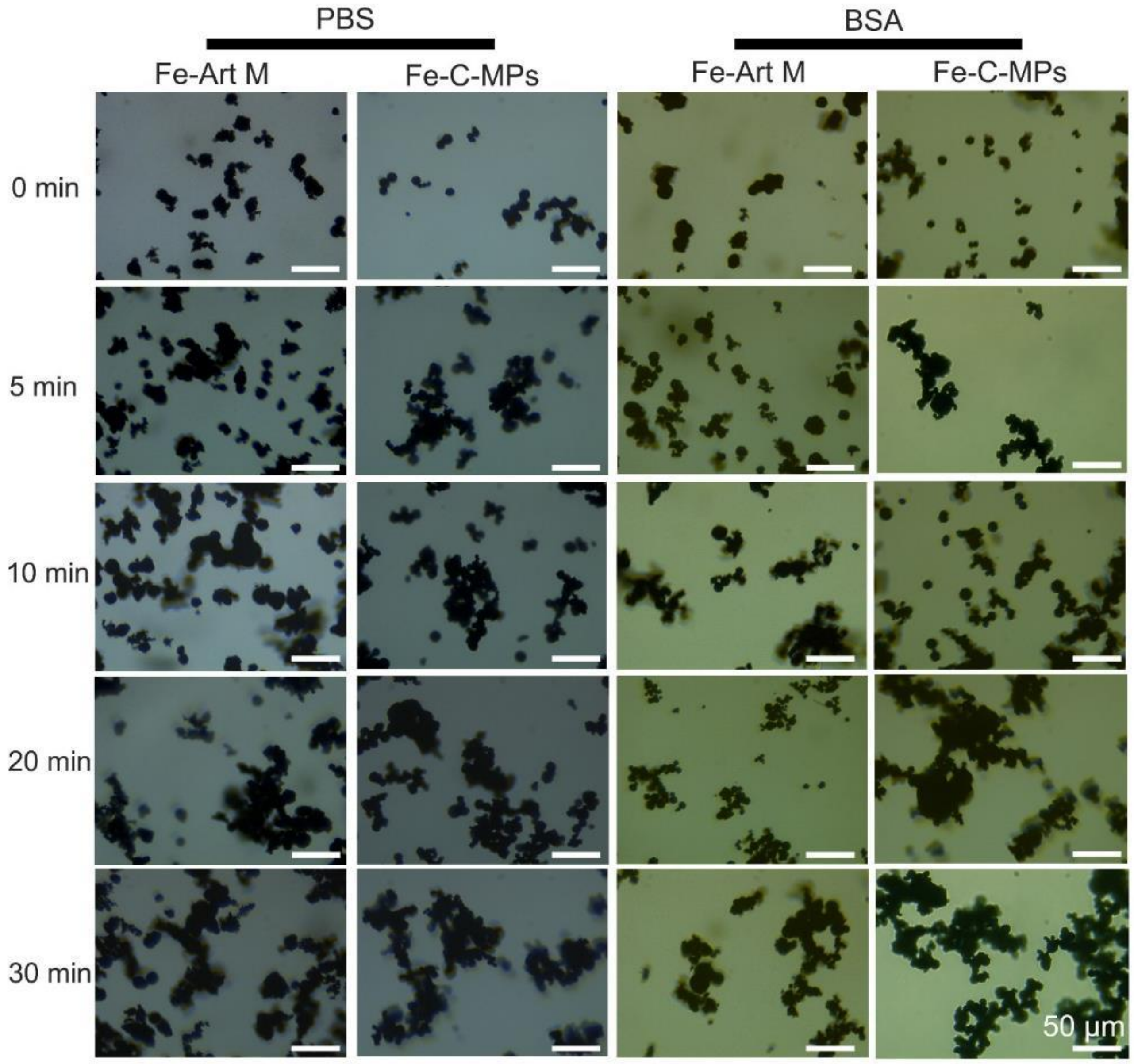

Supplementary Figure 7. The optical microscope images of Fe-Art M and Fe-C-MPs in PBS and BSA-contained solutions after standing for varied durations. Experiments were repeated independently three times with similar results. 0 min indicates that the samples are just finishing the shaking, and there are no obvious aggregates.

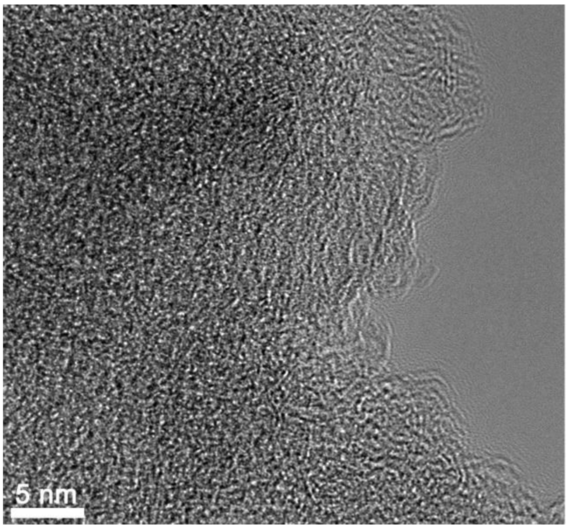

Supplementary Figure 8. HRTEM images of Fe-Art M. Experiments were repeated independently three times with similar results.

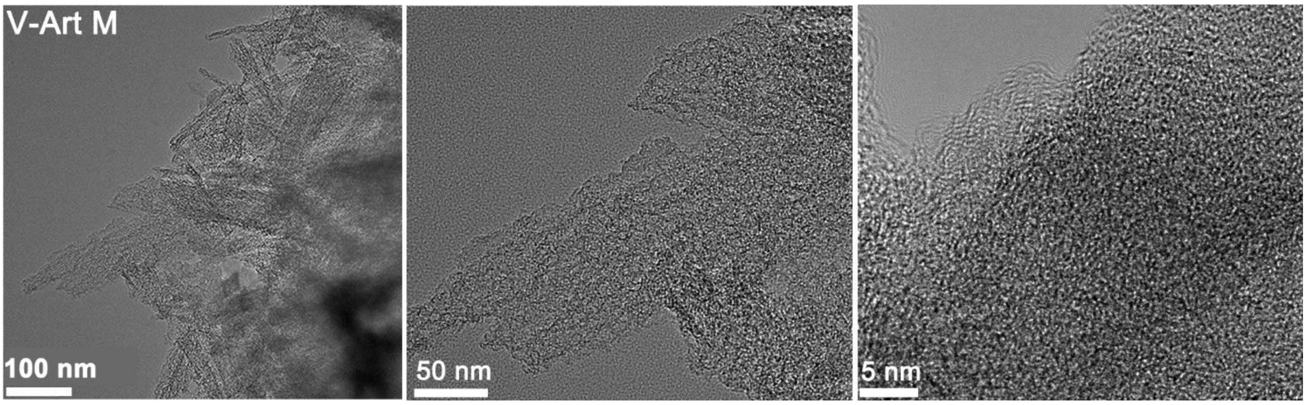

Supplementary Figure 9. HRTEM images of V-Art M. Experiments were repeated independently three times with similar results.

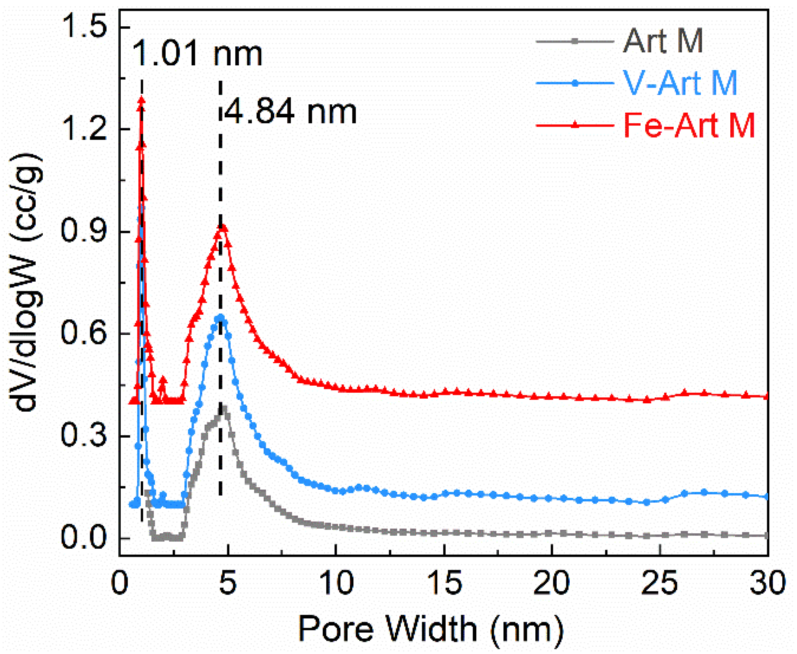

Supplementary Figure 10. Pore distribution of Art M, V-Art M, and Fe-Art M by BET method.

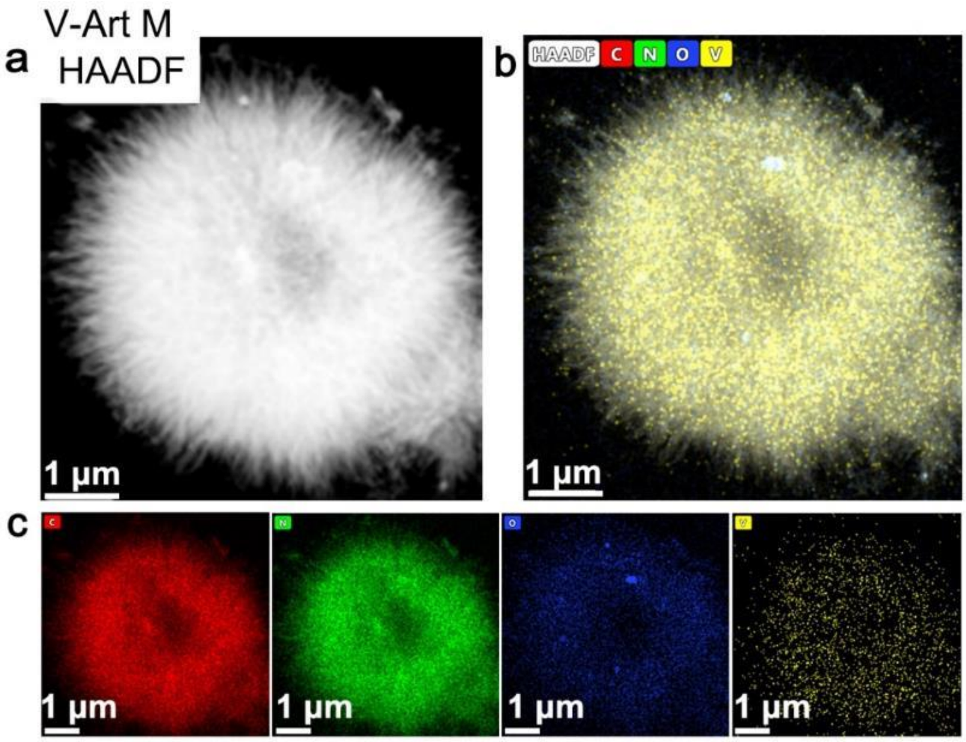

Supplementary Figure 11. **a** The high-angle annular dark-field scanning transmission electron microscopy (HAADF-STEM) images and **b-c** EDX mapping of V-Art M. Experiments were repeated independently **a**, **b**, **c** three times with similar results.

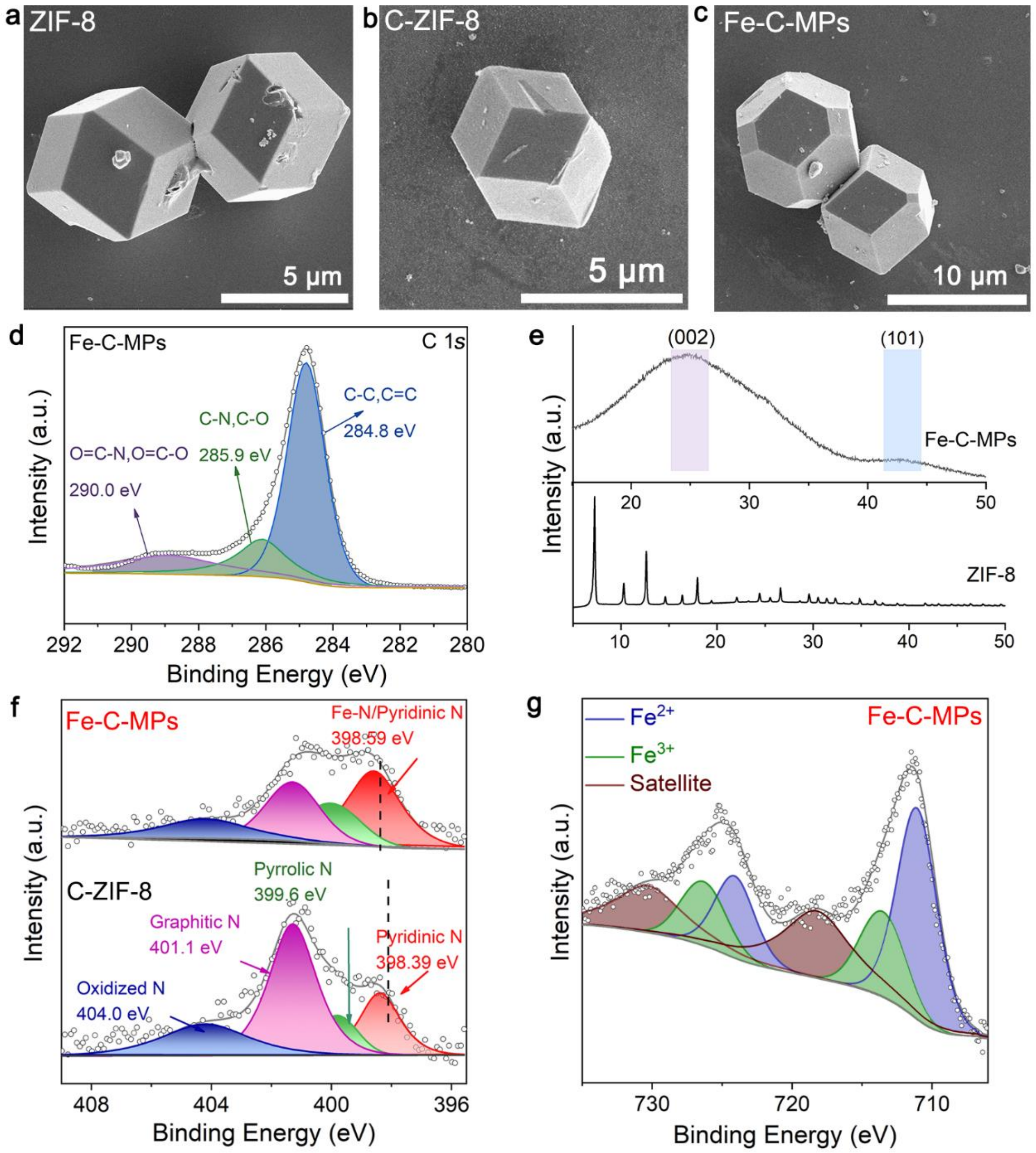

Supplementary Figure 12. SEM images of **a** ZIF-8 large crystal, **b** carbonized ZIF-8 (C-ZIF-8), and **c** Fe-C-MPs. Experiments were repeated independently **a**, **b**, **c** three times with similar results. **d** High-resolution XPS spectra of C 1*s* in Fe-C-MPs. **e** XRD patterns of ZIF-8 and Fe-C-MPs. **f** Comparison of curve-fitted high-resolution XPS N 1*s* spectra of the C-ZIF-8 and Fe-C-MPs. **g** High-resolution Fe 2*p* XPS spectra for Fe-C-MPs. The SEM images of ZIF-8, C-ZIF-8, and Fe-C-MPs demonstrates that they share a similar siz as Art Ms. As can be seen in the XRD and XPS results, the metal Fe in Fe-C-MPs exists in the form of coordination structure due to no peaks of iron oxides and the peak shift after the coordination with Fe, indicating the chemical structure of Fe-C-MPs is similar to that of Fe-Art M.

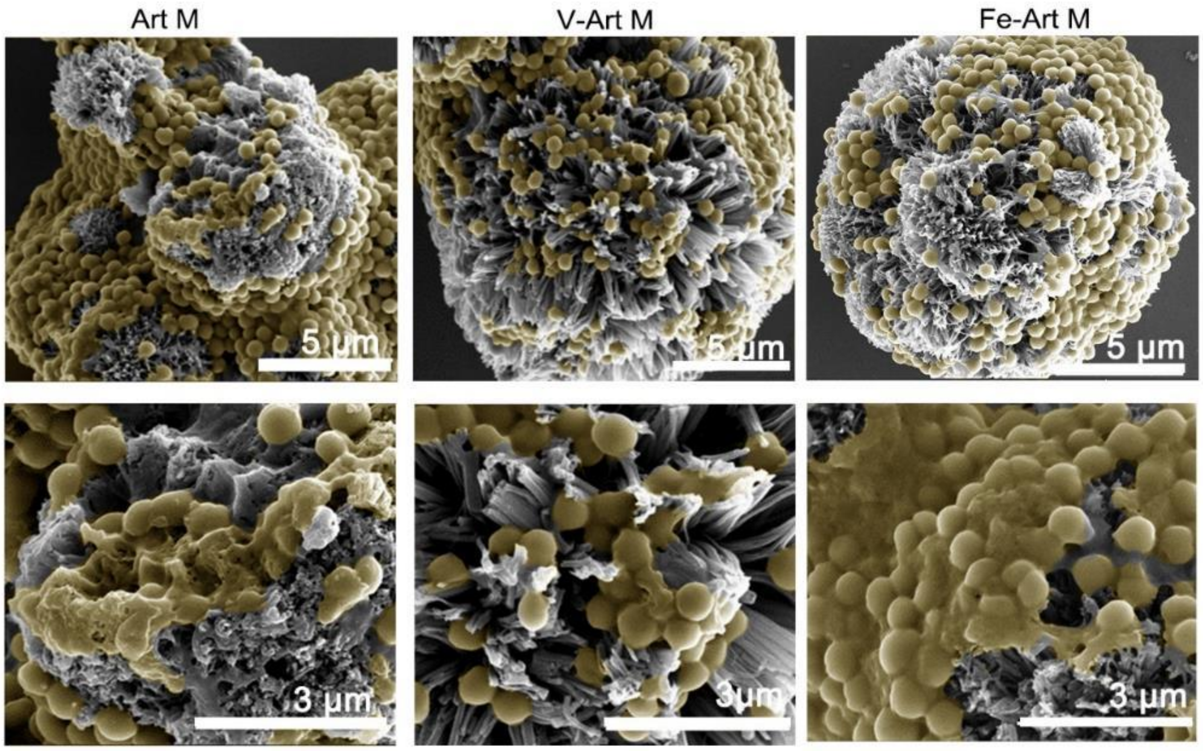

Supplementary Figure 13. SEM images of Art M, V-Art M, and Fe-Art M treated with MRSA. Experiments were repeated independently per group three times with similar results.

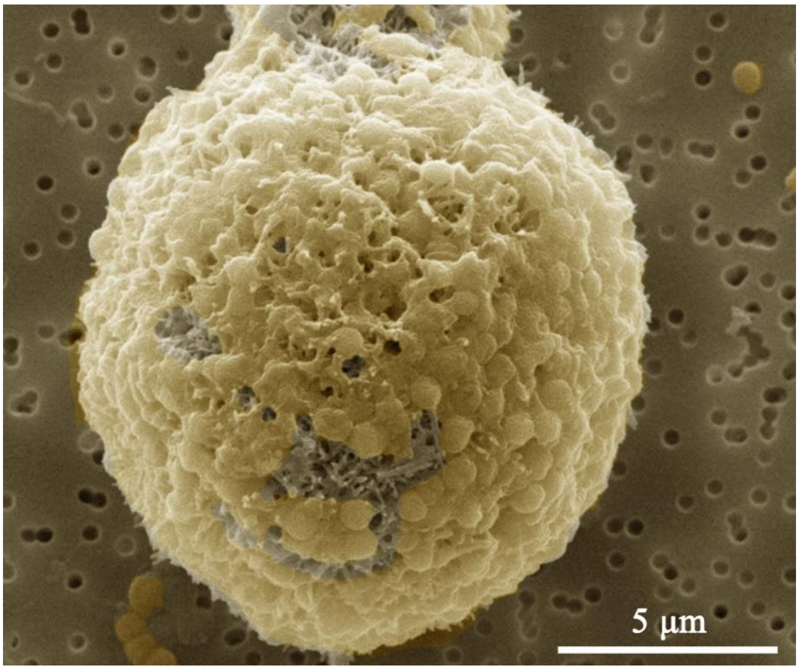

Supplementary Figure 14. Cryo-SEM image of Fe-Art M treated with MRSA. Experiments were repeated independently three times with similar results.

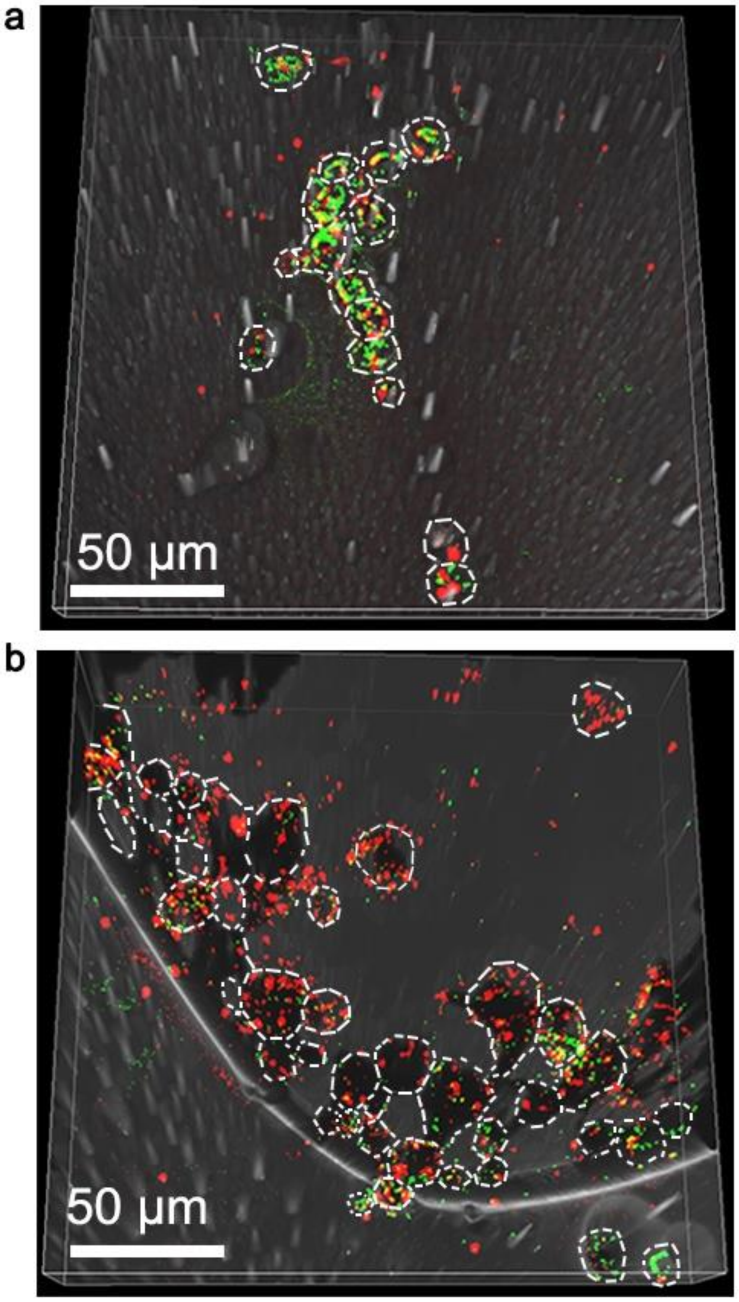

Supplementary Figure 15. 3D reconstructions from CLSM images of **a** V-Art M and **b** Fe-Art M when treated with MRSA. The V/Fe-Art M are marked with white lines. Experiments were repeated independently three times with similar results.

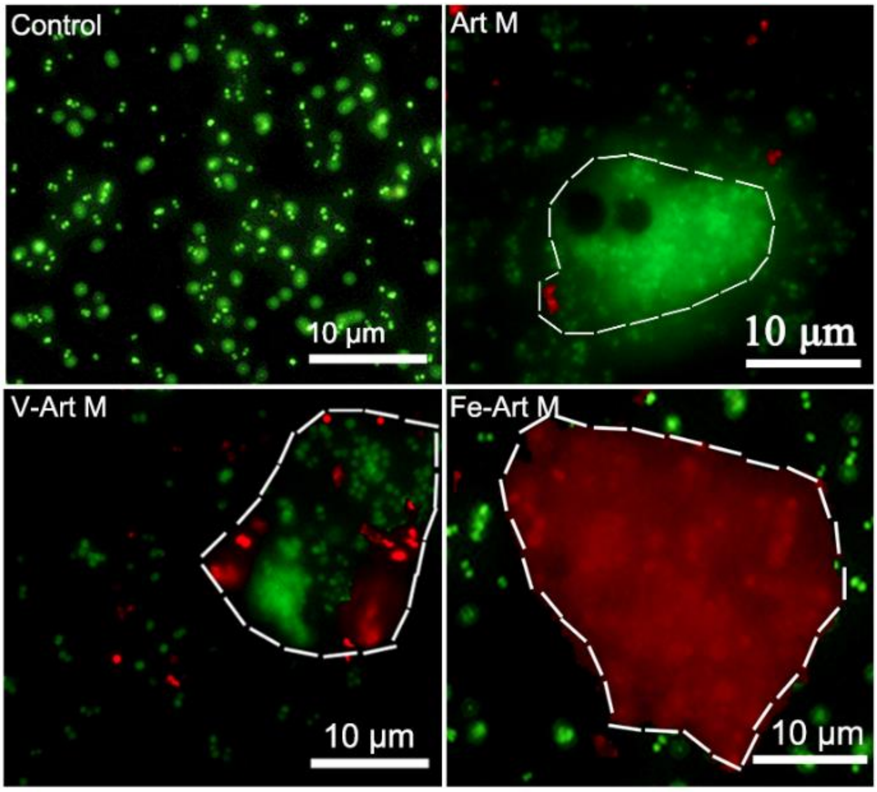

Supplementary Figure 16. Fluorescence microscopy images of Control, Art M, V-Art M and Fe-Art M when incubated with MRSA. Experiments were repeated independently per group three times with similar results.

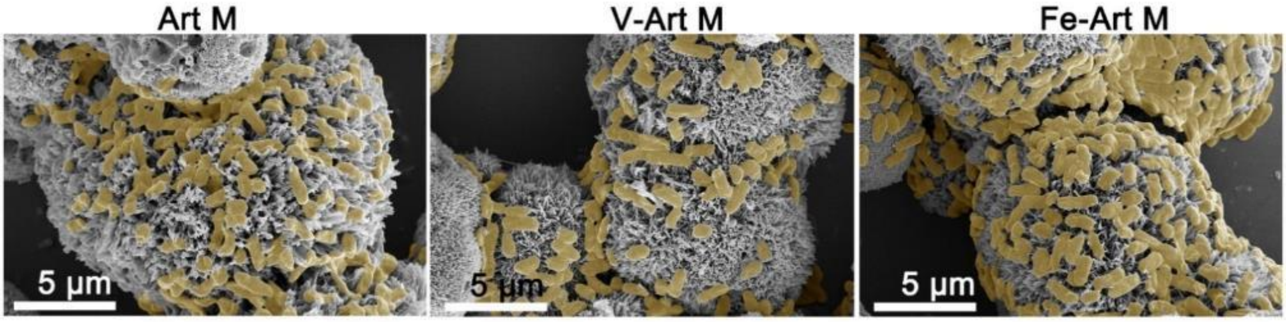

Supplementary Figure 17. SEM images of bacterial capture capability of Art Ms when incubated with extended-spectrum β-lactamase-producing *E. coli*. Experiments were repeated independently per group three times with similar results.

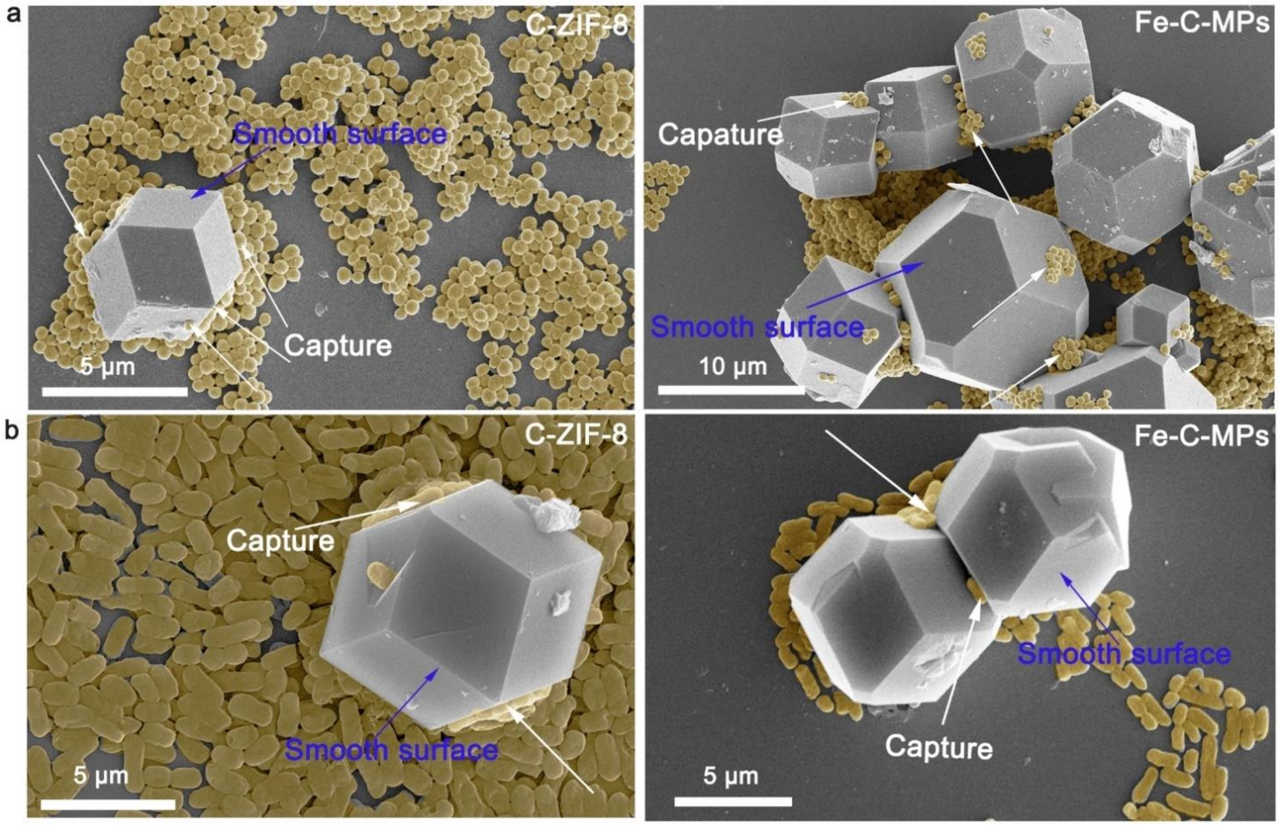

Supplementary Figure 18. SEM images of **a** MRSA and **b** extended-spectrum β-lactamase-producing *E. coli* after incubation with C-ZIF-8 and Fe-C-MPs (experiments were repeated independently **a**, **b** per group three times with similar results), which indicate that the carbon material with a smooth surface shows weak bacterial capture capability.

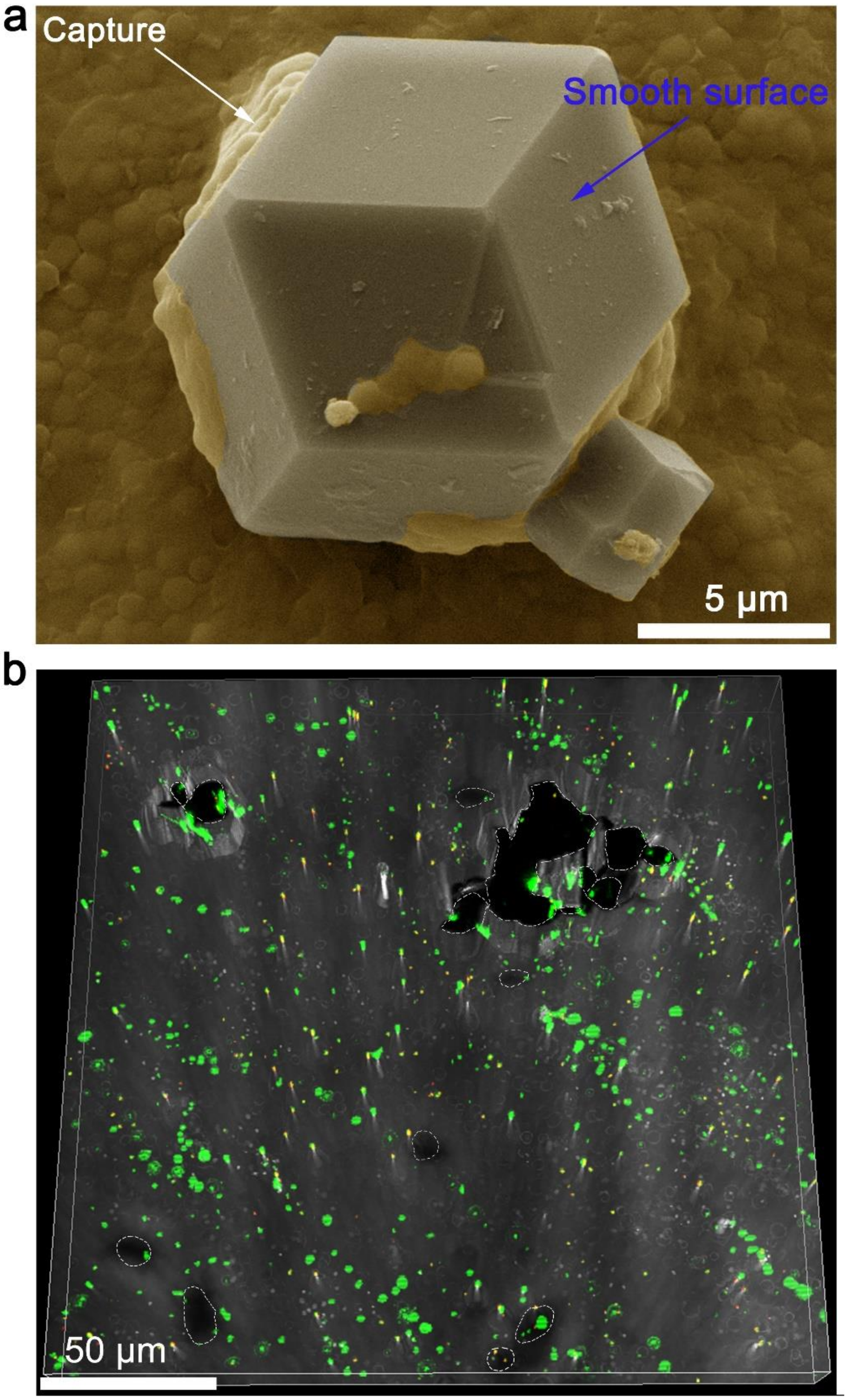

Supplementary Figure 19. **a** Cryo-SEM image of MRSA after incubation with Fe-C-MPs. **b** 3D reconstruction from CLSM image of Fe-C-MPs when treated with MRSA. The Fe-C-MPs are marked with white lines. Experiments were repeated independently **a**, **b** three times with similar results.

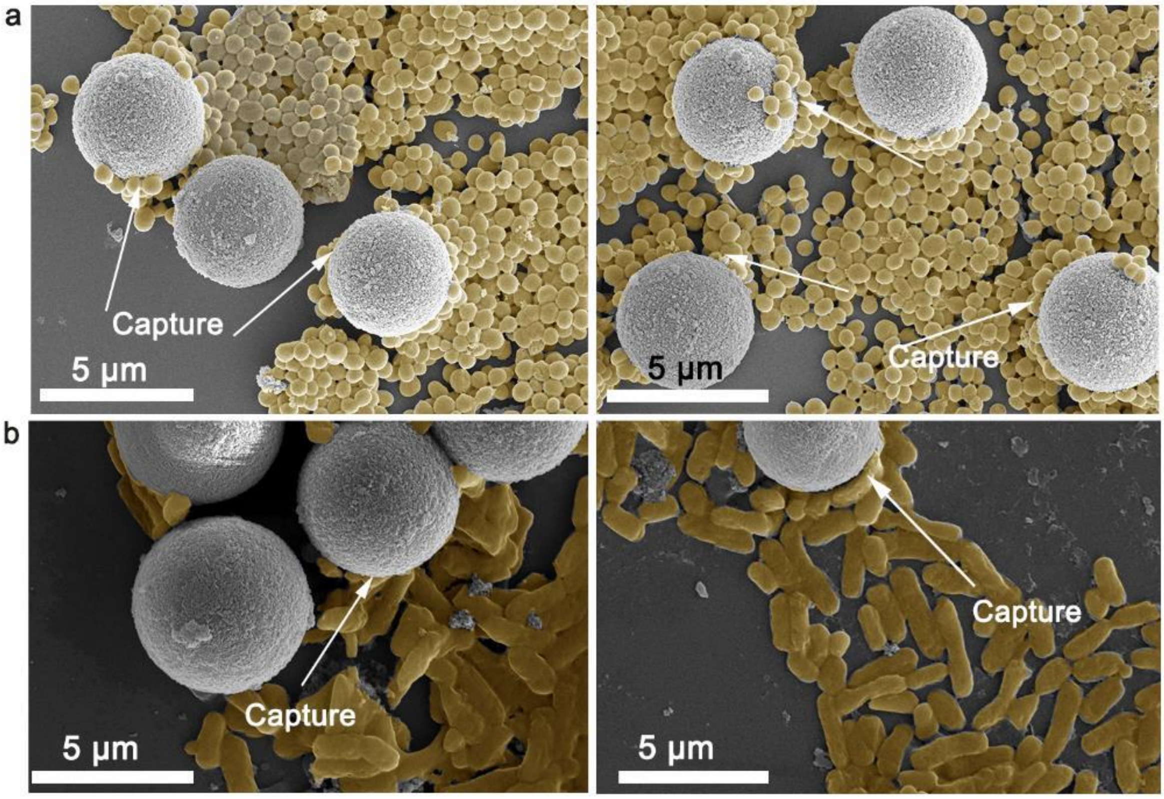

Supplementary Figure 20. SEM images of **a** MRSA and **b** extended-spectrum β-lactamase-producing *E. coli* after incubation with SiO_2 microspheres (experiments were repeated independently **a**, **b** three times with similar results), which indicates that the slightly rough surface exhibits relatively limited bacterial capture capability.

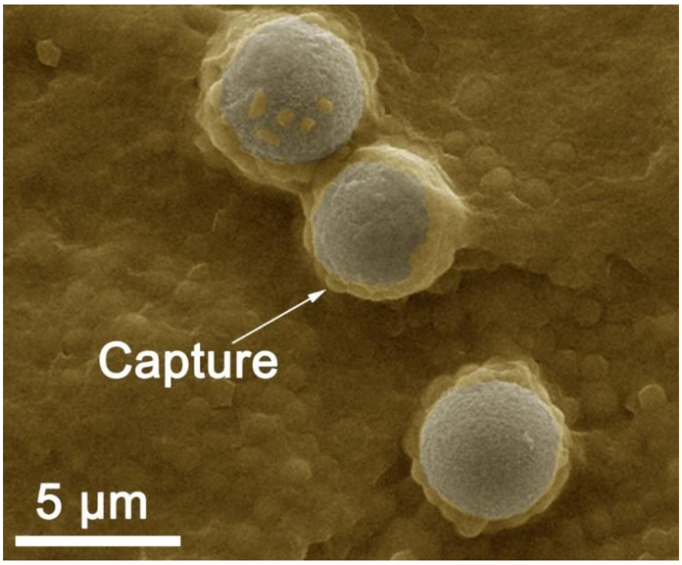

Supplementary Figure 21. Cryo-SEM images of MRSA after incubation with SiO_2 microspheres. Experiments were repeated independently three times with similar results.

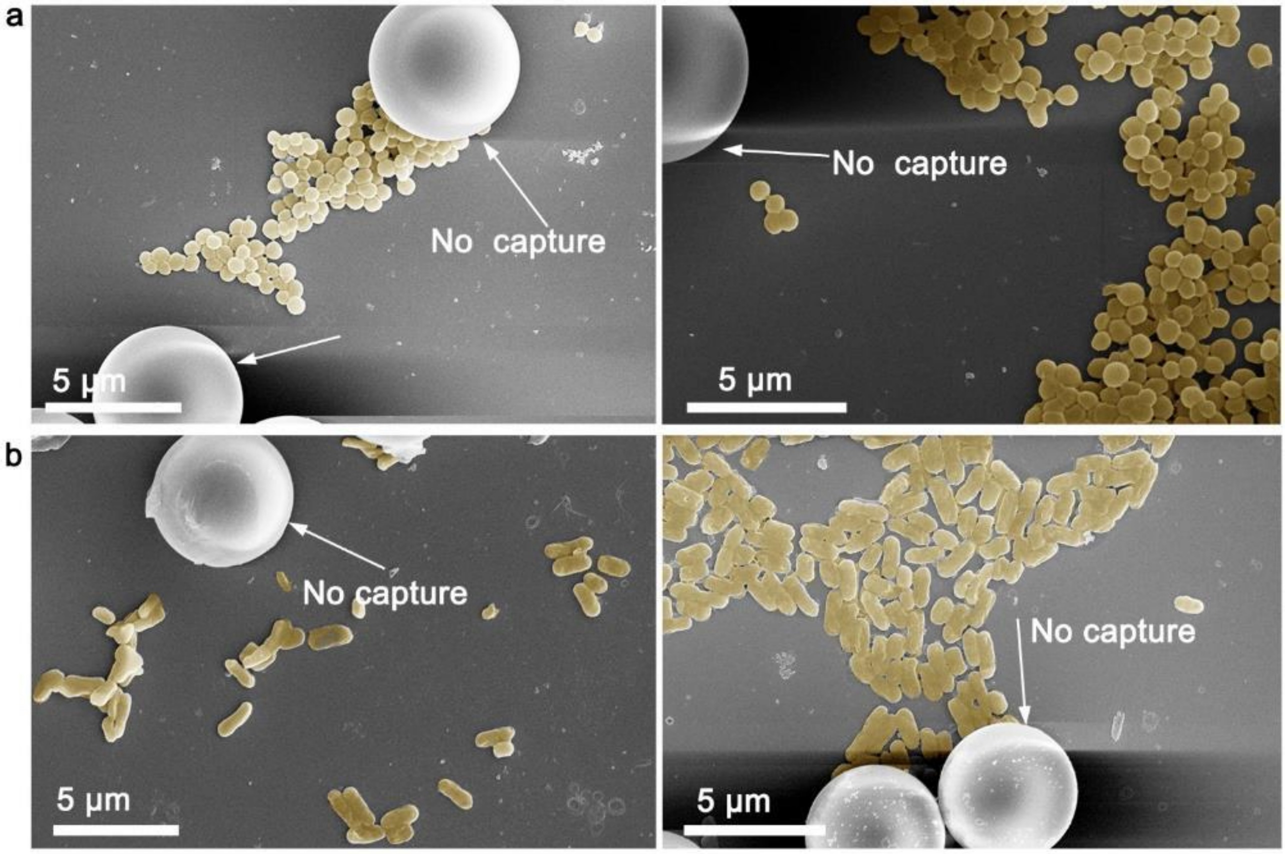

Supplementary Figure 22. SEM images of **a** MRSA and **b** extended-spectrum β-lactamase-producing *E. coli* after incubation with polystyrene microspheres (experiments were repeated independently **a**, **b** three times with similar results), which indicate that the polymeric material with a smooth surface shows nearly no bacterial capture capability.

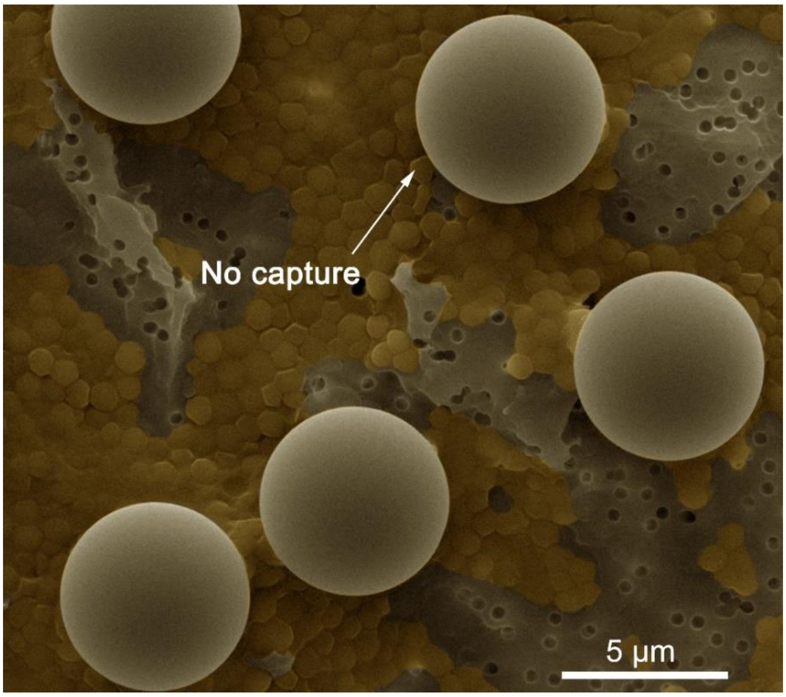

Supplementary Figure 23. Cryo-SEM images of MRSA after incubation with polystyrene microspheres (experiments were repeated independently three times with similar results).

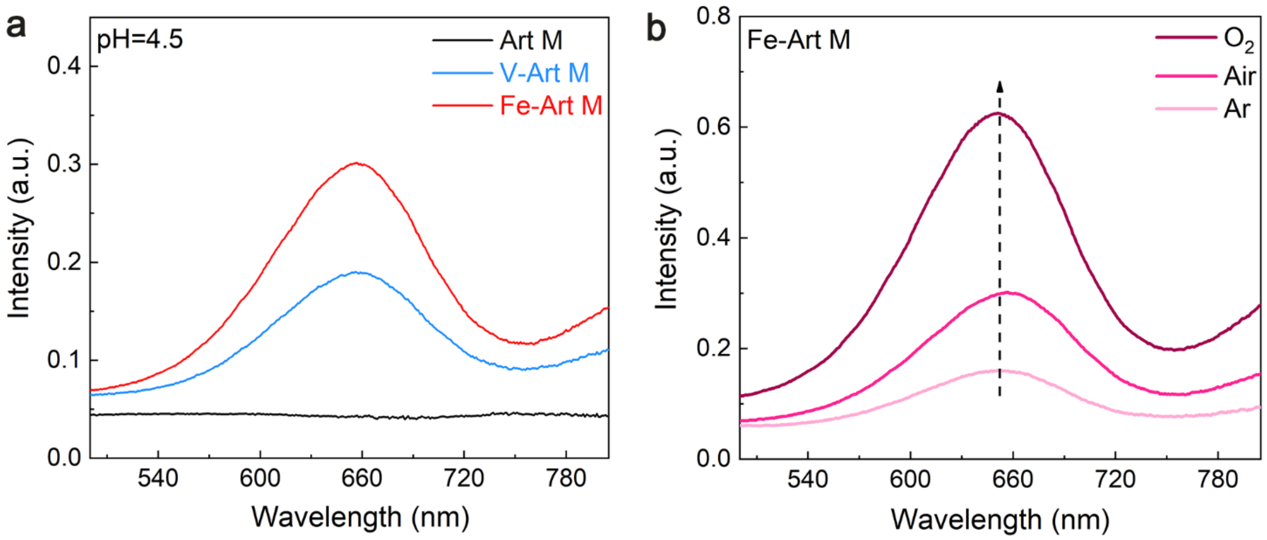

Supplementary Figure 24. The oxidase-like activity: **a** Typical absorption spectra of TMB after catalytic oxidation in pH 4.5 acetate buffer. **b** Ultraviolet-visible (UV-vis) absorption spectra of TMB after catalytic oxidation in pH 4.5 by the Fe-Art M in O_2-saturated, air-saturated, and Ar-saturated sodium acetate–acetic acid buffer. In **a**, **b**, a.u. indicates the arbitrary units.

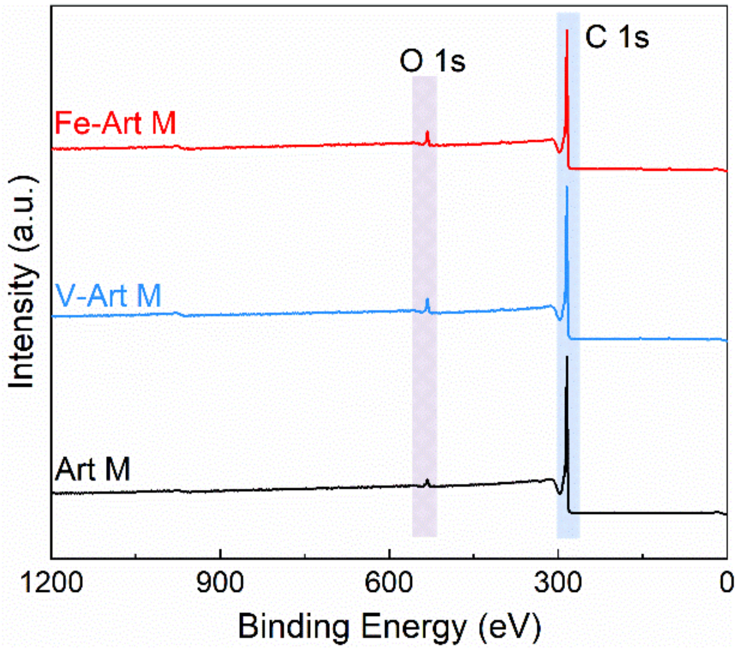

Supplementary Figure 25. XPS survey curves of Art M, V-Art M, and Fe-Art M. The a.u. indicates the arbitrary units.

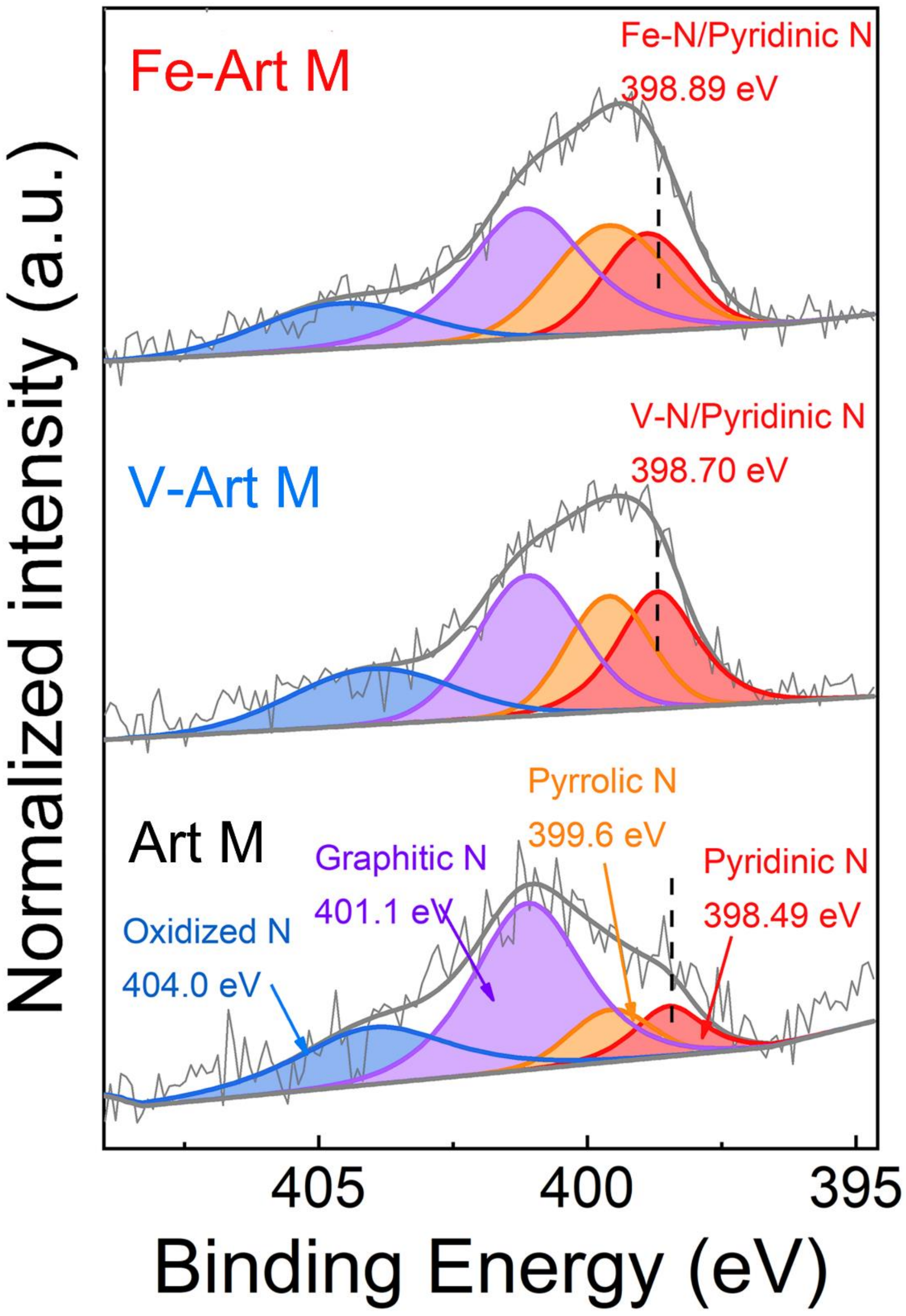

Supplementary Figure 26. Comparison of curve-fitted high-resolution XPS N 1*s* spectra of Art M, V-Art M, and Fe-Art M (a.u. indicates the arbitrary units). There is a peak shift of pyridinic N in V-Art M (398.70 eV) when compared to that in Art M (398.49 eV), indicating the formation of V-N_x/pyridinic-N[1].

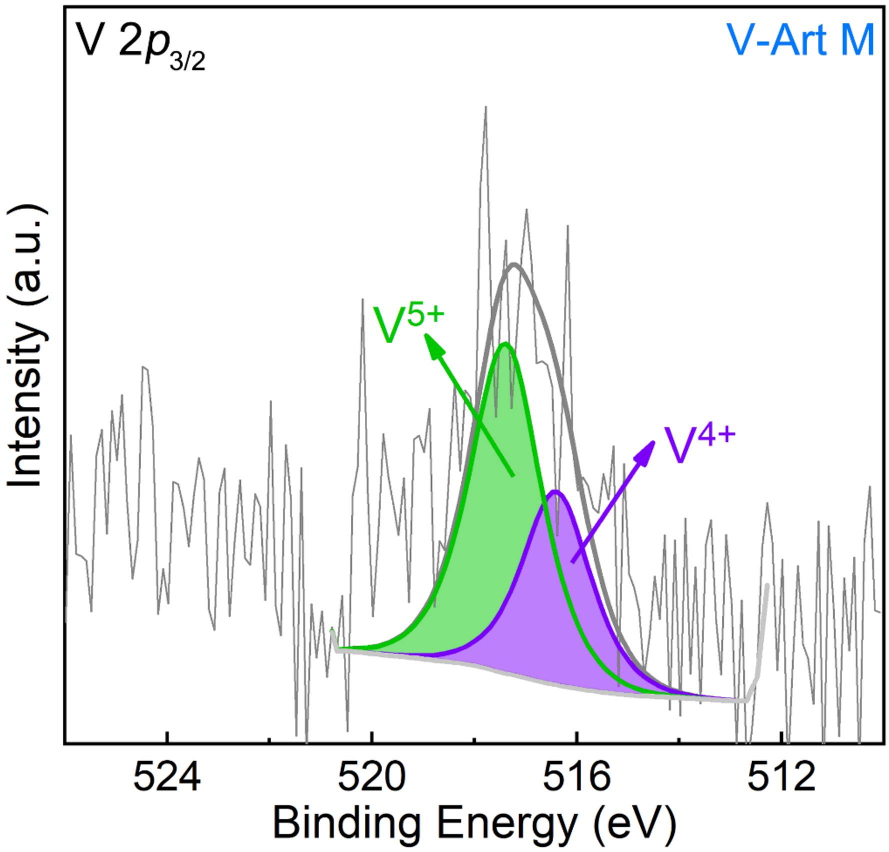

Supplementary Figure 27. High-resolution V 2*p* XPS spectra for V-Art M (a.u. indicates the arbitrary units).

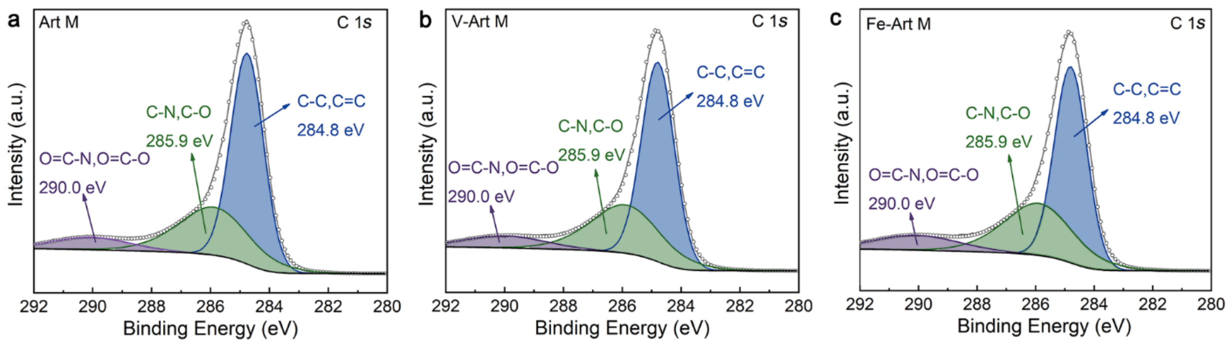

Supplementary Figure 28. High-resolution XPS spectra of C 1*s* in **a** Art M, **b** V-Art M, and **c** Fe-Art M, and in **a**, **b**, **c**, a.u. indicates the arbitrary units.

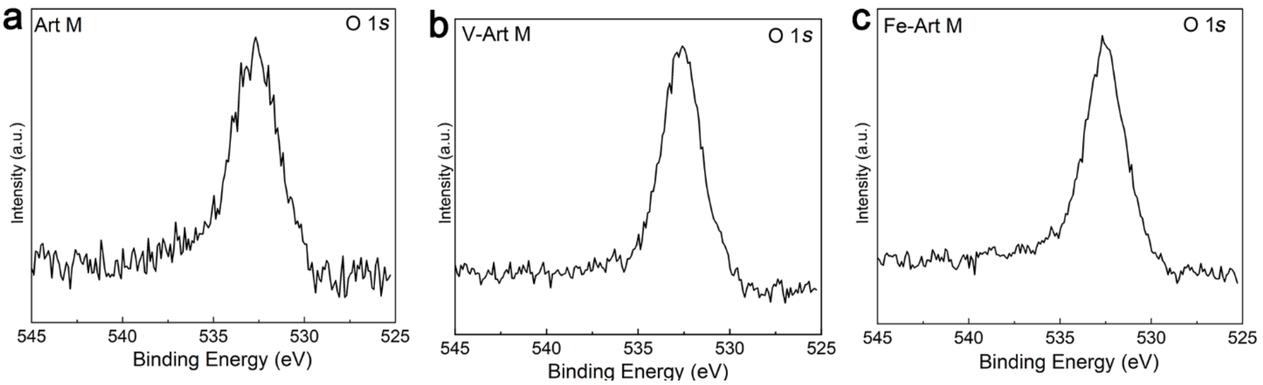

Supplementary Figure 29. High-resolution XPS spectra of O 1*s* in **a** Art M, **b** V-Art M, and **c** Fe-Art M, and in **a**, **b**, **c**, a.u. indicates the arbitrary units.

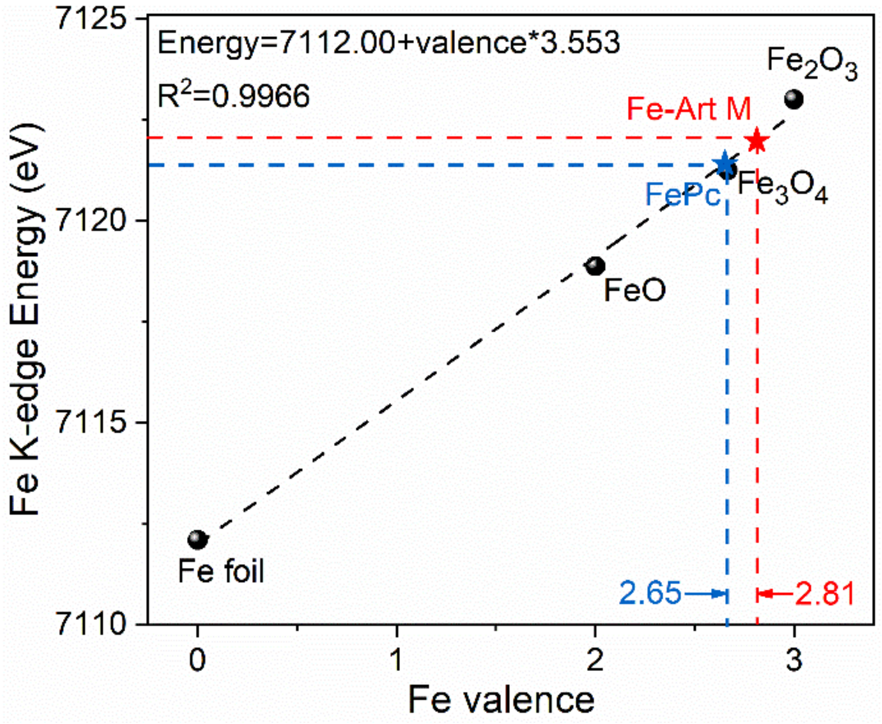

Supplementary Figure 30. Relation between the Fe K-edge absorption energy (E_0) and valence states for Fe-Art M and reference materials.

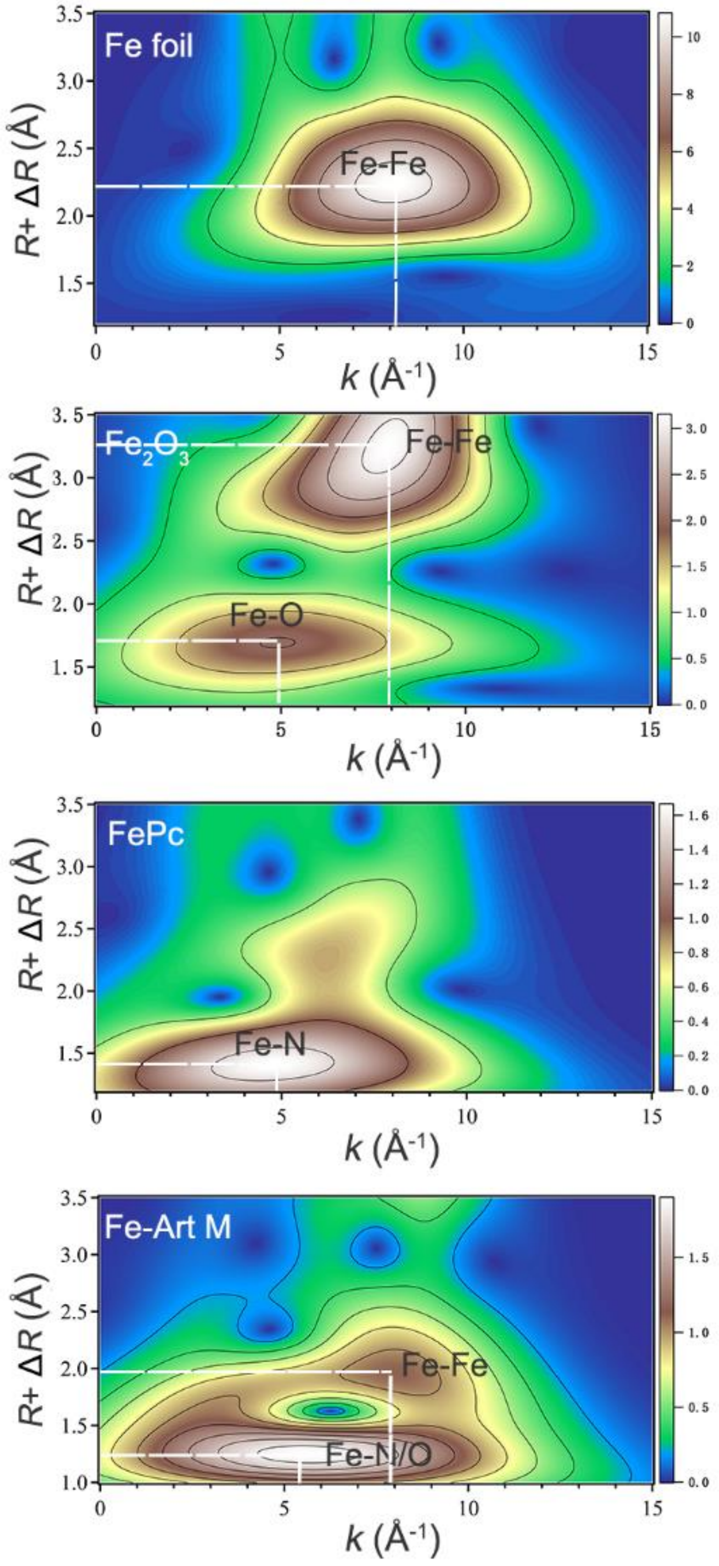

Supplementary Figure 31. WT-EXAFS of Fe foil, Fe_2O_3, FePc, and Fe-Art M.

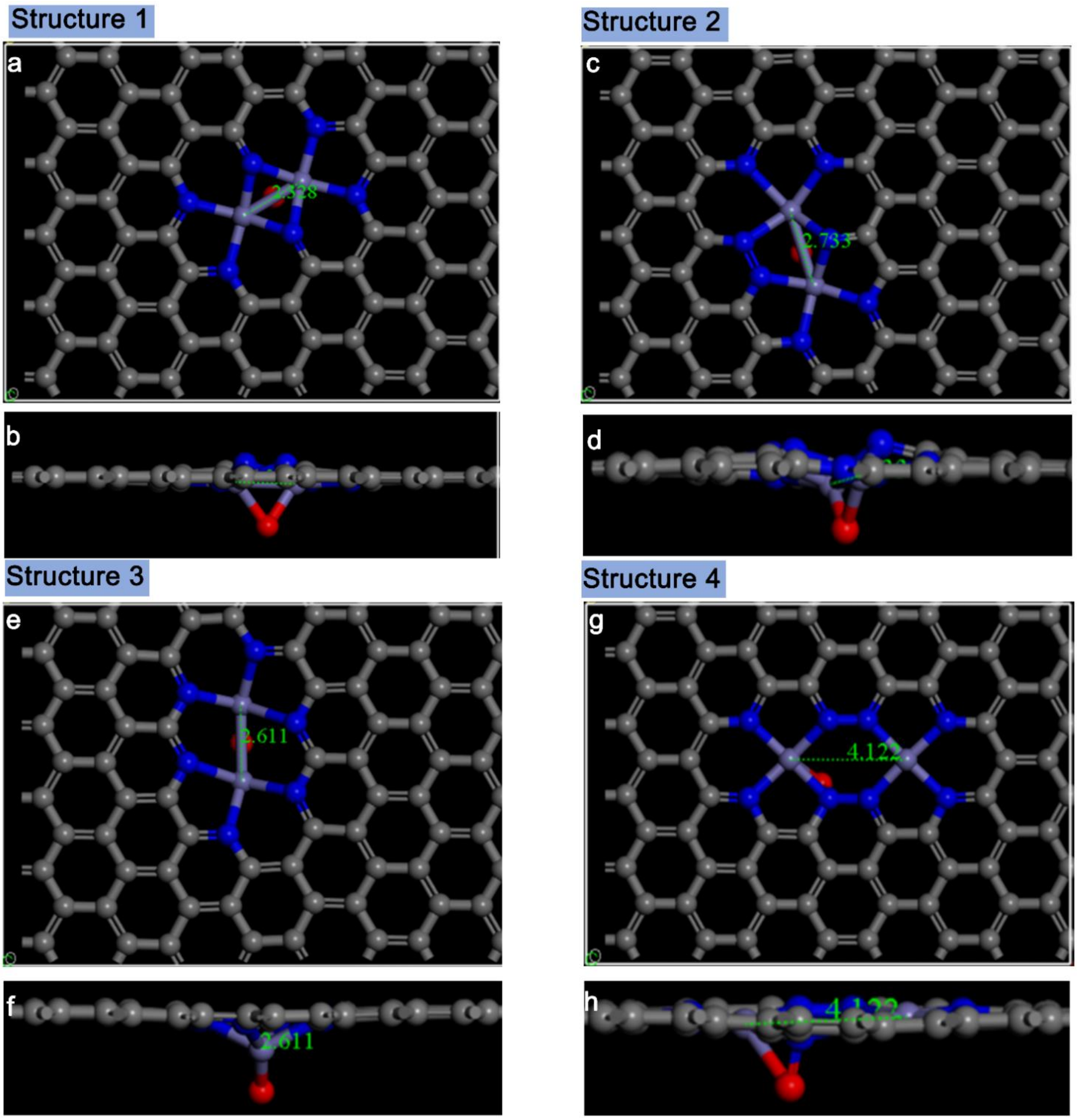

Supplementary Figure 32. Speculated four kinds of chemical structure models and the corresponding Fe-Fe bond lengths**. a, c, e, g,** top views**. b, d, f, h,** side views**.**

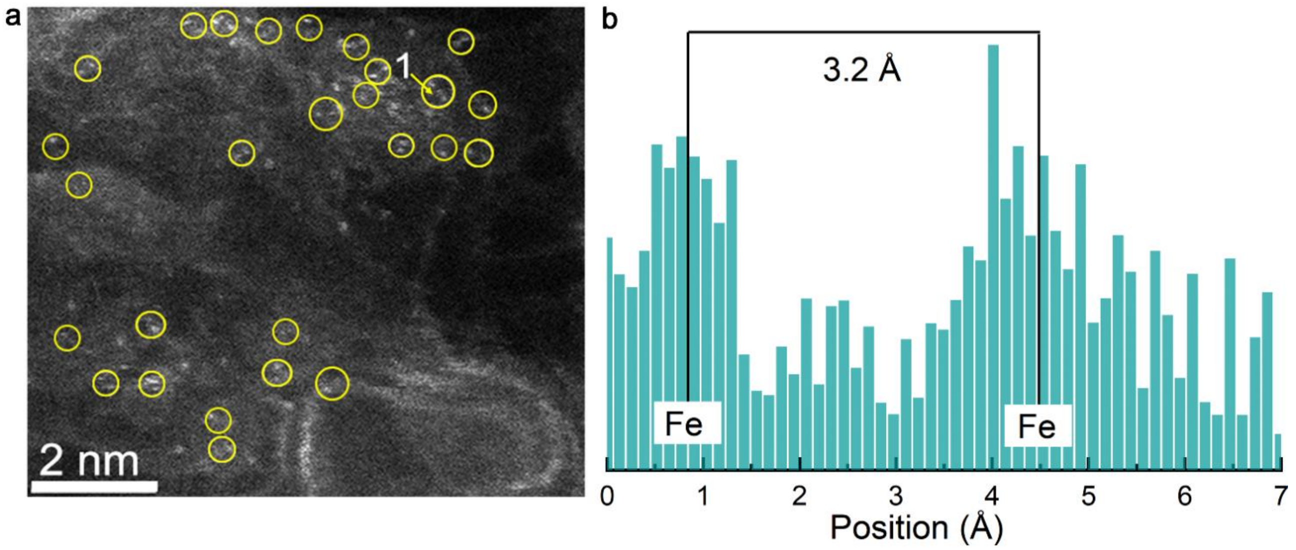

Supplementary Figure 33. a High-resolution HAADF-STEM image of Fe-Art M. Experiments were repeated independently three times with similar results. **b** Intensity profile obtained in zone 1 shows the Fe-Fe projection distance on the visual plane. It should be noticed that a minority of Fe-Fe with a large bond length of about 3.2 Å have also been detected, which indicates that some other minority Fe-N structures are also formed in our Fe-Art M.

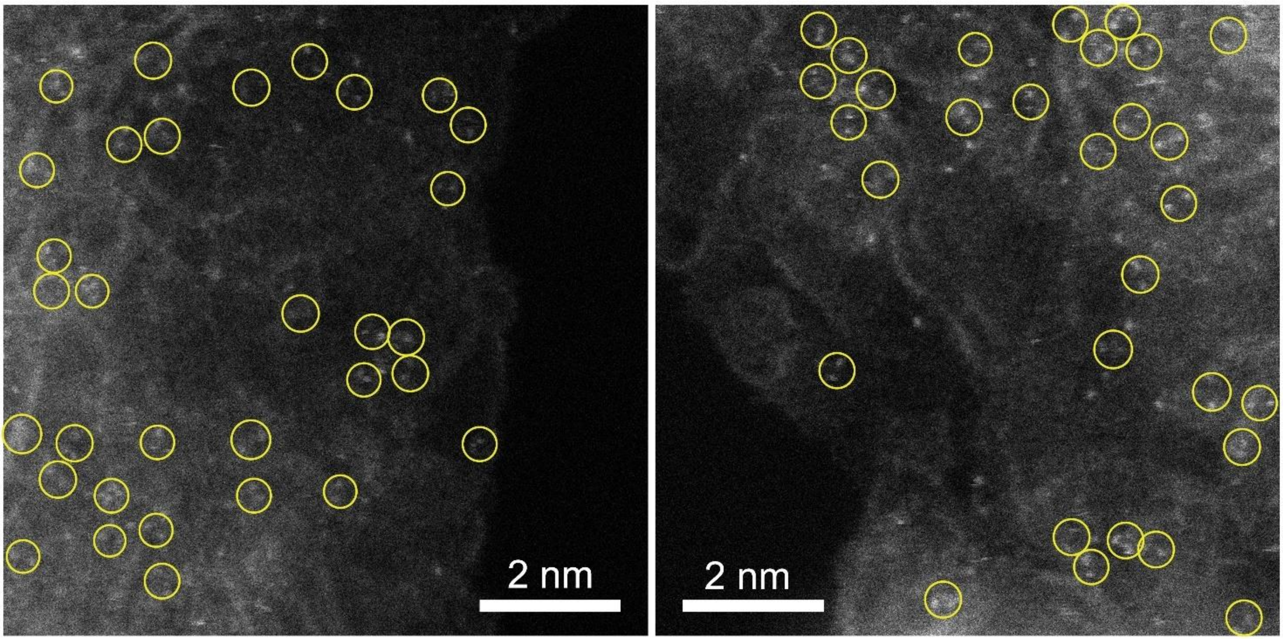

Supplementary Figure 34. High-resolution HAADF-STEM images of V-Art M. Experiments were repeated independently three times with similar results.

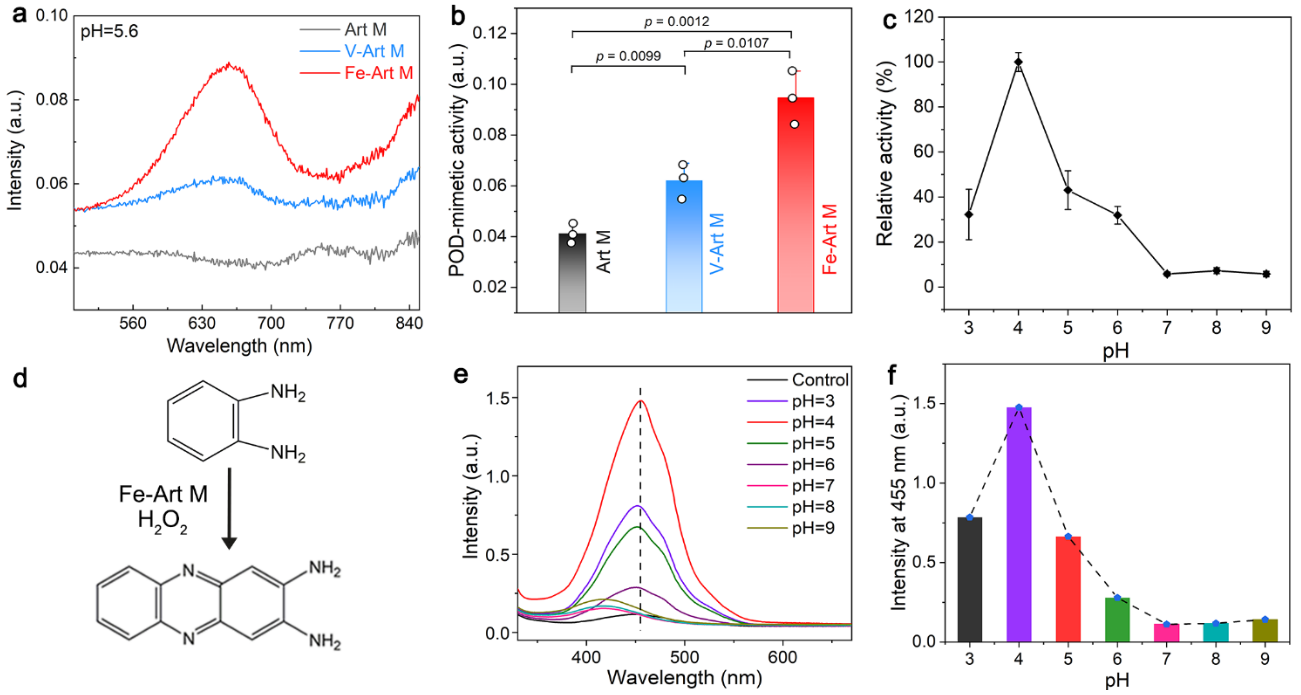

Supplementary Figure 35. a Typical absorption spectra of TMB after catalytic oxidation with H_2O_2 in pH 5.6 acetate buffer. **b** Intensity at 652 nm of different samples (n=3 independent experiments, data are presented as mean ± SD). **c** pH-dependent absorbance changes at 652 nm by using Fe-Art M as POD-mimics (n=3 independent experiments, data are presented as mean ± SD). **d** Illustration of the reaction of o-phenylenediamine (OPD), **e, f** Fe-Art M show POD-like activity in a pH-dependent manner, indicating that the Fe-Art M can catalyze the oxidation of OPD to produce colorimetric reactions. In **b**, *p* values are assessed by unpaired Student's two-side t tests. In **a**, **b**, **e**, **f**, a.u. indicates the arbitrary units.

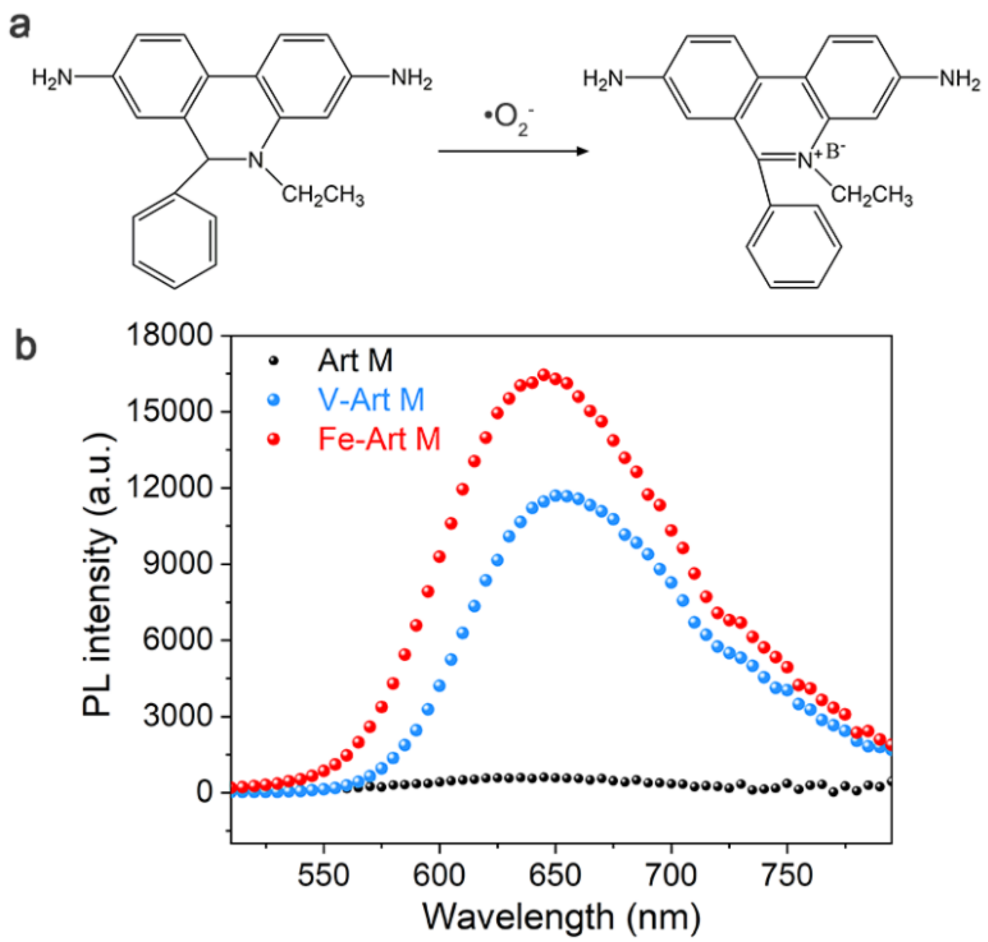

Supplementary Figure 36. a Illustration of the reaction of hydroethidine (HE) by $•O_2^-$. **b** The fluorescence spectra of HE after reaction with the catalytic produced $•O_2^-$ by different samples, and a.u. indicates the arbitrary units.

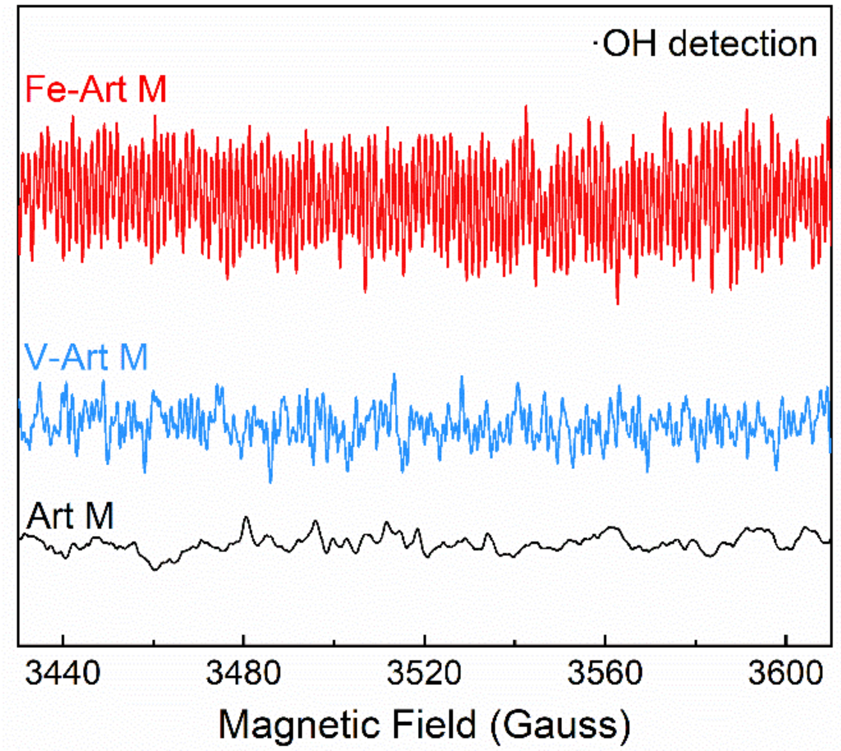

Supplementary Figure 37. EPR spectra for detection of •OH generated from different systems in HOAc/NaOAc solution by using DMPO as trapping agent.

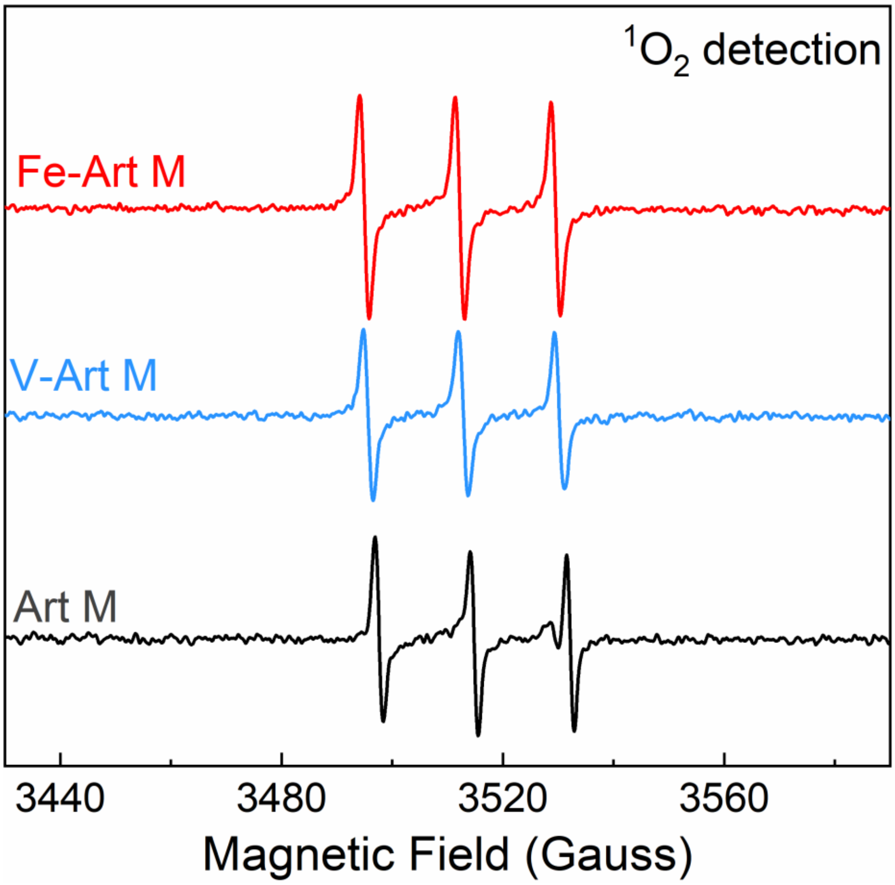

Supplementary Figure 38. The EPR spectra of the in situ $^1O_2$ radical detection by TEMP.

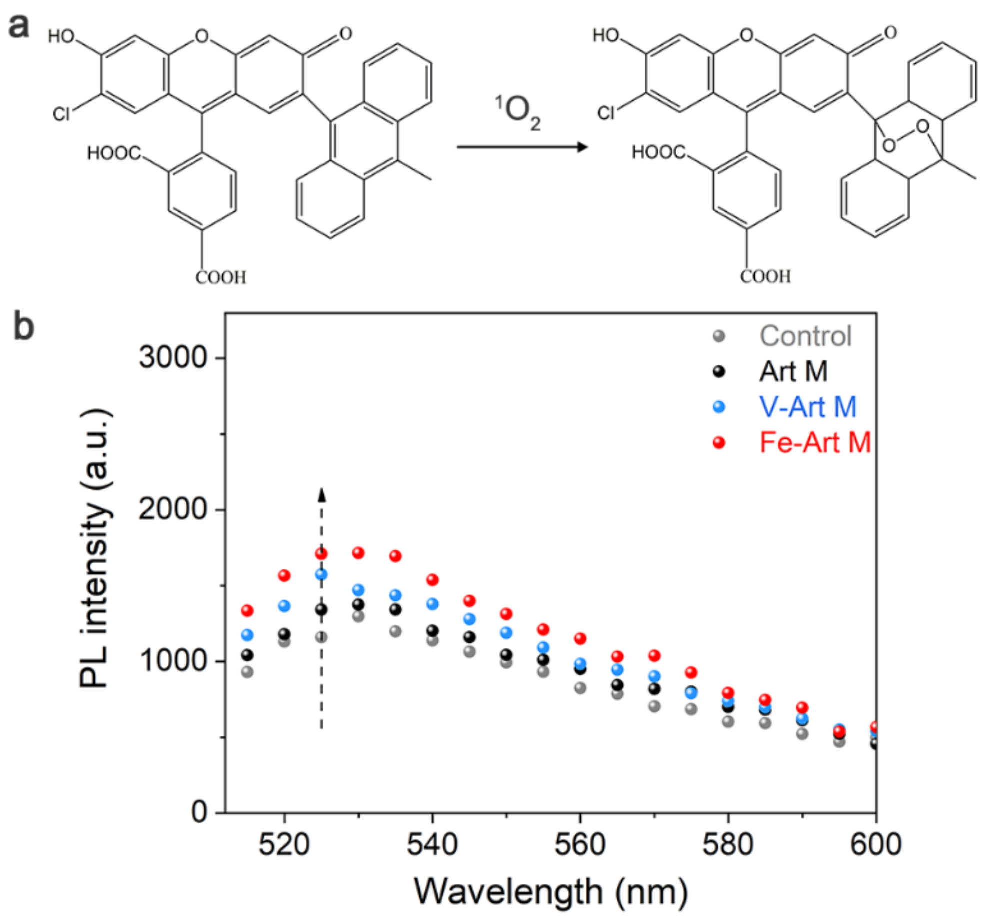

Supplementary Figure 39. a Formation of the endoperoxide upon the reaction of singlet oxygen sensor green (SOSG) with $^1O_2$. **b** The fluorescence spectra of SOSG after reaction with $^1O_2$ with different samples, and a.u. indicates the arbitrary units.

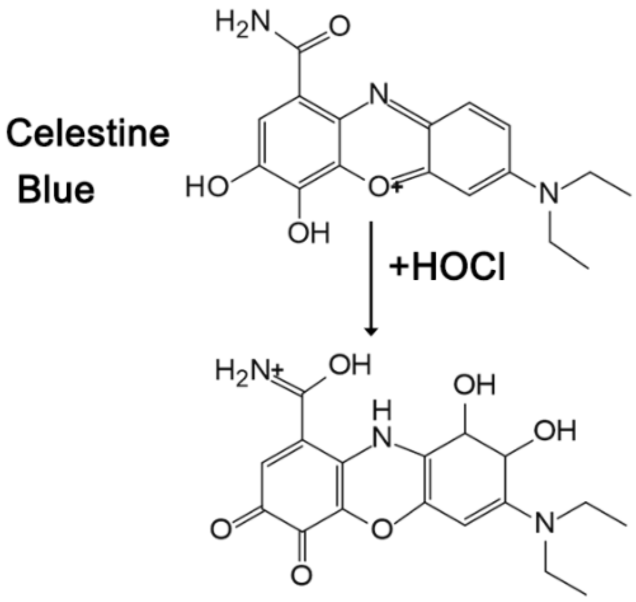

Supplementary Figure 40. Illustration of the oxidation of CB molecules by the catalytic produced HOCl.

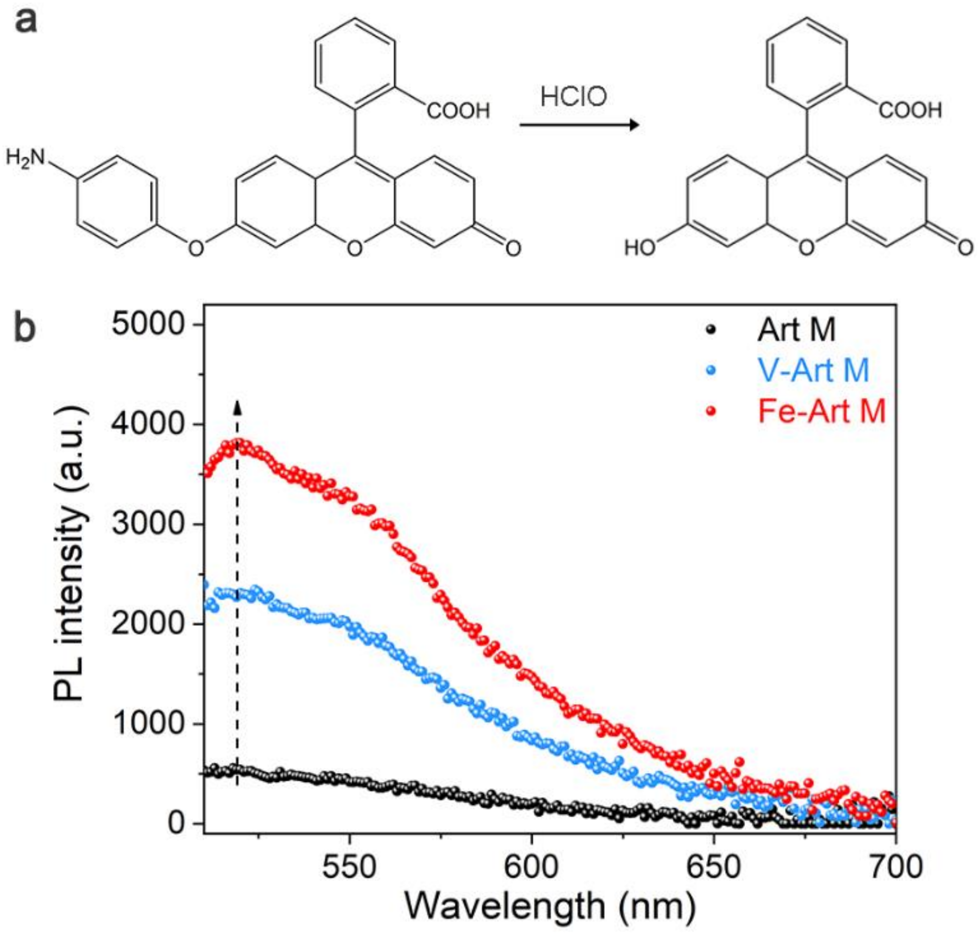

Supplementary Figure 41. a Illustration of the chemical reaction of aminophenyl fluorescein (APF) by HOCl. **b** The fluorescence spectra of APF after reaction with HClO produced by different samples, and a.u. indicates the arbitrary units.

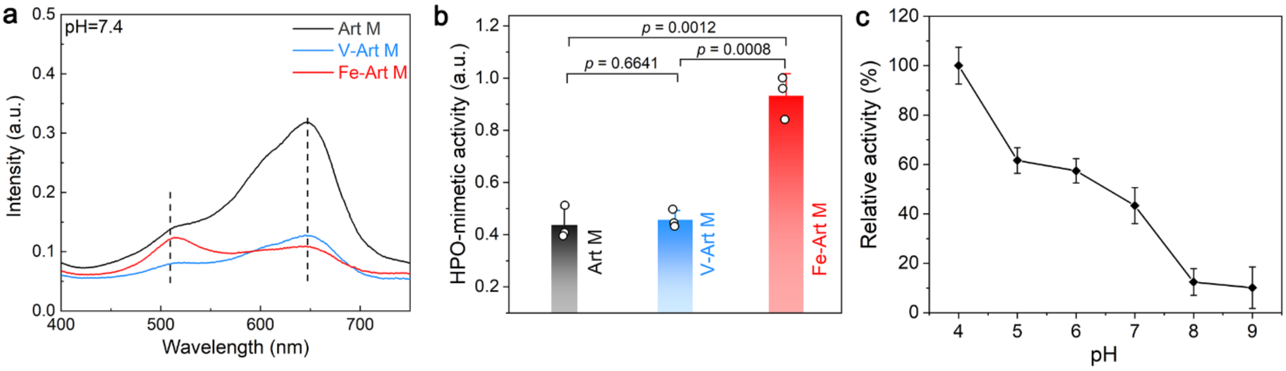

Supplementary Figure 42. **a** Detection of activity by using celestine blue as agents for haloperoxidase-mimic activity in pH 7.4 acetate buffer. **b** Specific value of intensity at 512 nm and 648 nm (n=3 independent experiments, data are presented as mean ± SD). **c** pH-dependent absorbance changes by using Fe-Art M as HPO-mimics, it should be noticed that the CB chromogenic reaction is not stable below pH 3;[2] thus, we only measure the reaction starting from pH 4 to pH 9 (n=3 independent experiments per group, data are presented as mean ± SD). In **b**, *p* values are assessed by unpaired Student's two-side t tests. In **a**, **b**, a.u. indicates the arbitrary units.

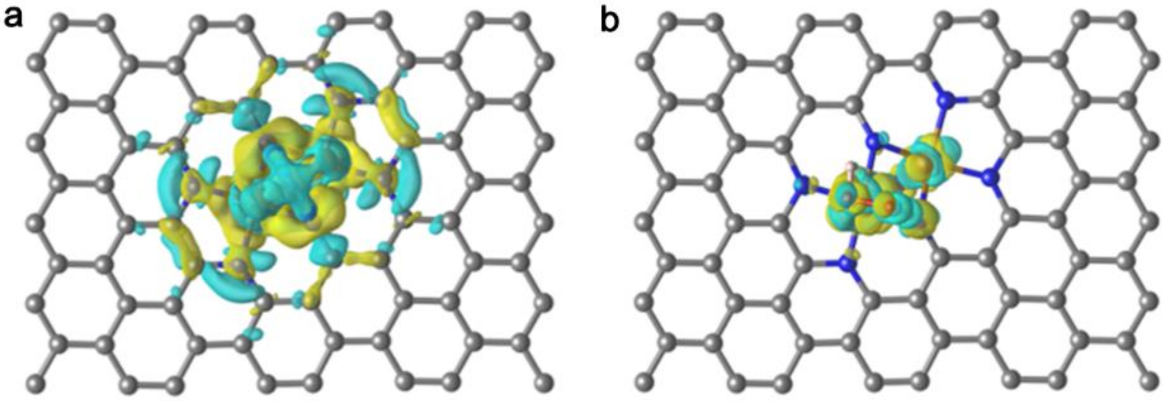

Supplementary Figure 43. Electron density difference plot of **a** Fe_2N_6O and **b** Fe_2N_6O with adsorbed H_2O_2. Yellow contours indicate electron accumulation, and light green contours denote electron deletion.

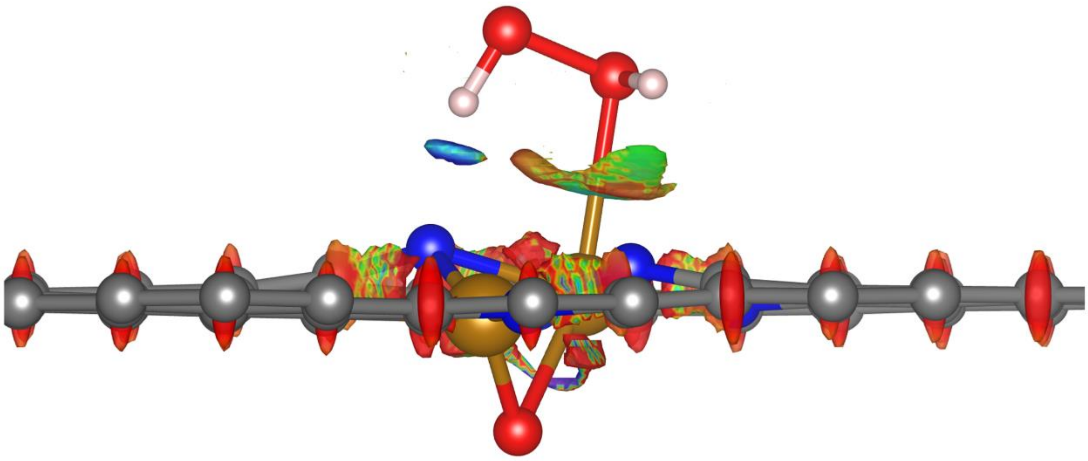

Supplementary Figure 44. Reduced density gradient (RGD) of Fe_2N_6O with adsorbed H_2O_2 molecules. The acting force between N and H is the hydrogen bond, and the acting force between Fe and O is Van der Waals action.

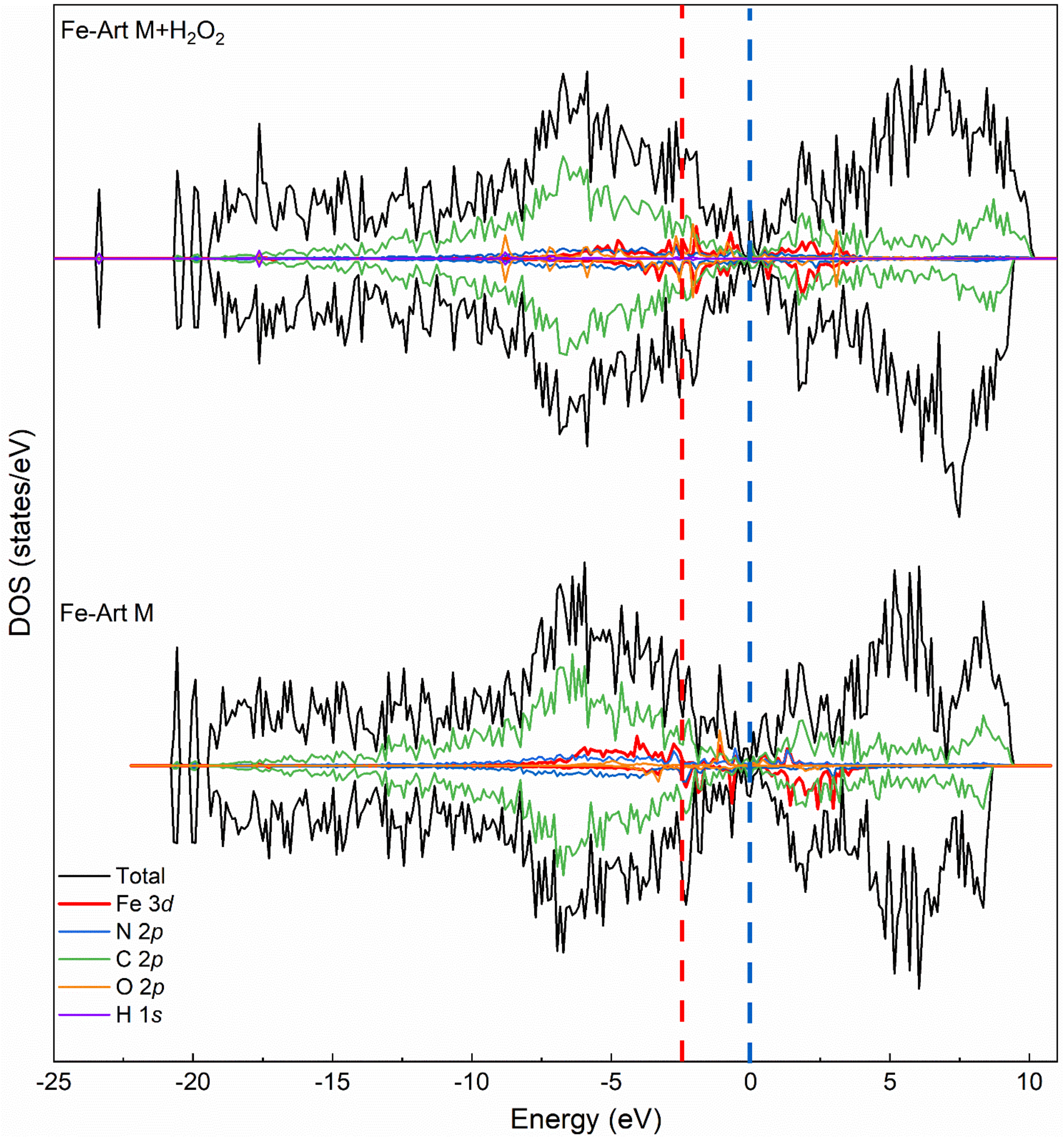

Supplementary Figure 45. Projected density of states (PDOS) of the Fe_2N_6O and Fe_2N_6O with adsorbed H_2O_2 molecules. The zero-energy corresponds to the vacuum level.

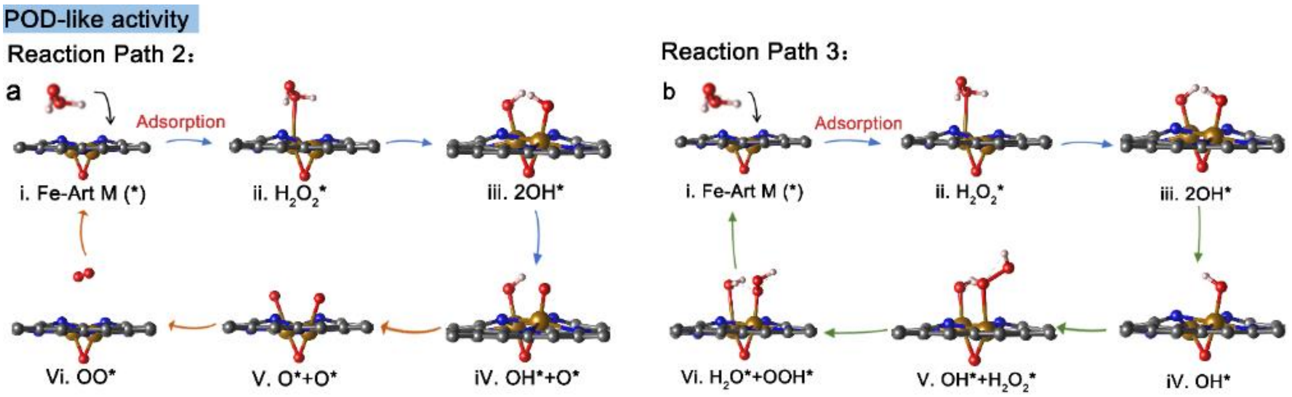

Supplementary Figure 46. Proposed other reaction mechanism for Fe-Art M catalyzing H_2O_2 in the generation of $•O_2^-$. Atom colors: orange, Fe; brown, C; gray, N; red, O; white, H; and green, Cl.

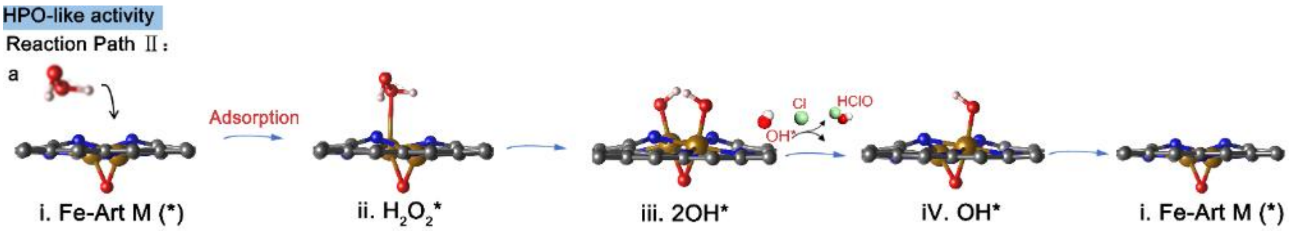

Supplementary Figure 47. Two proposed reaction mechanism schematics and the free energy diagrams of Fe-Art M Fenton reaction toward generating HClO as HPO-mimics. Atom colors: orange, Fe; brown, C; gray, N; red, O; white, H; and green, Cl.

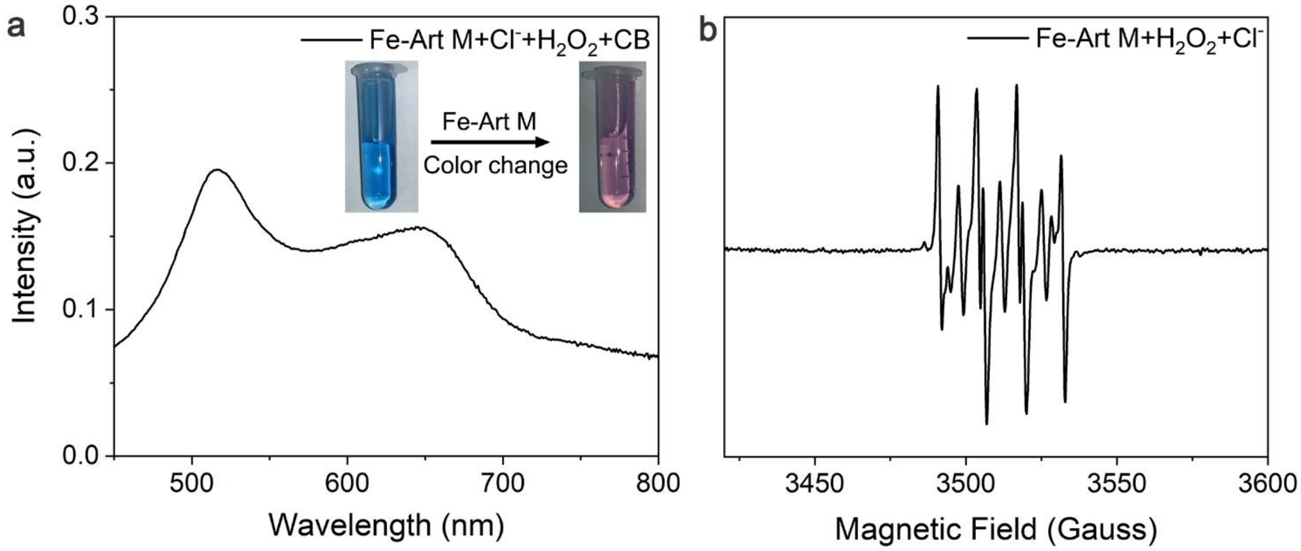

Supplementary Figure 48. **a** Detection of HClO. **b** DMPO spin-trapping EPR spectra for $\bullet O_2^-$ detection. In **a**, a.u. indicates the arbitrary units.

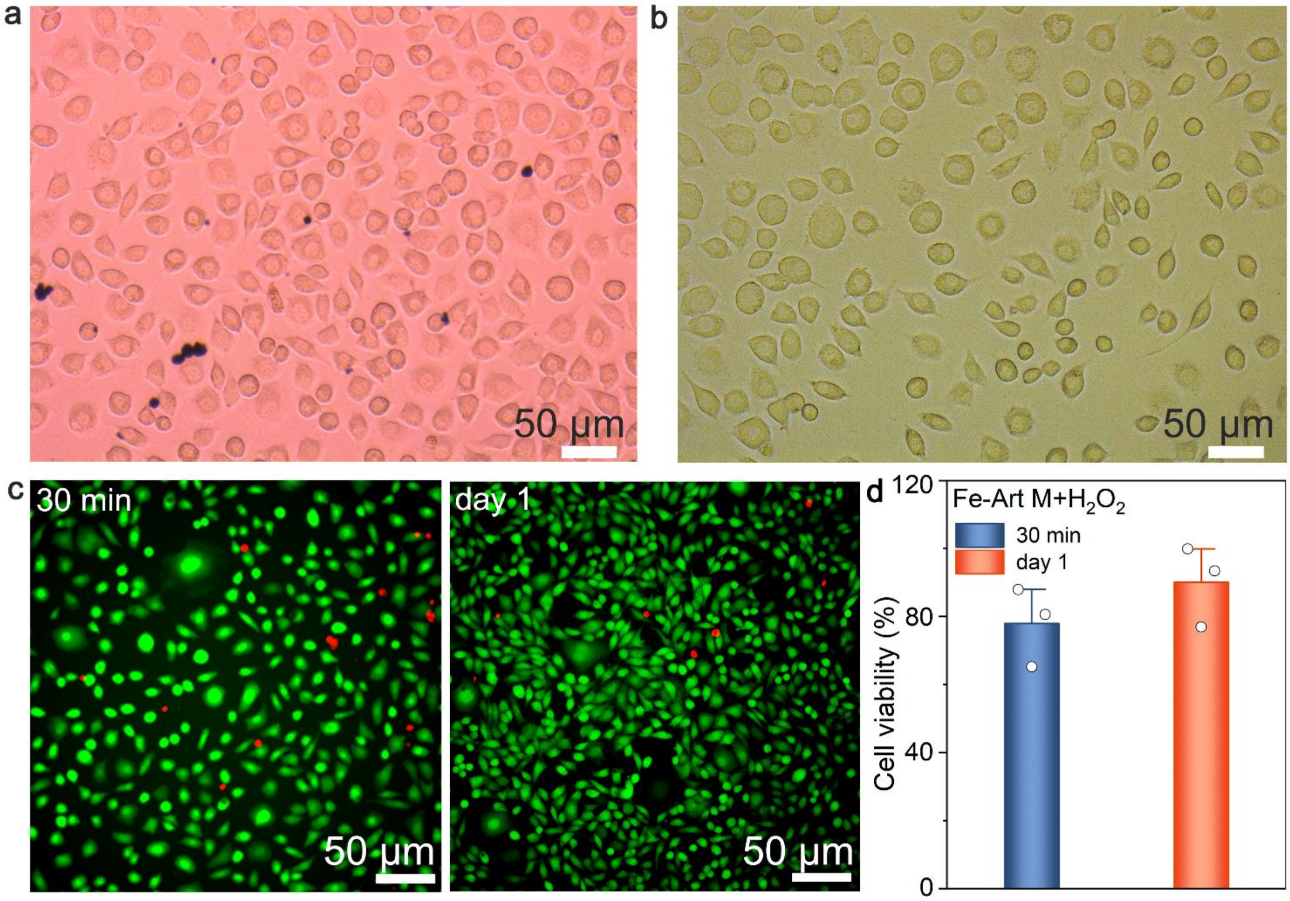

Supplementary Figure 49. The optical microscope of **a** adhered L929 cells when co-cultured with 10 μg/mL Fe-Art M and H_2O_2 (100 μM) for 30 min and **b** cells after removal of Fe-Art M by changing medium and washing with PBS solution twice. It is obvious to see that there is no Fe-Art M after washing. **c** Representative live/dead cell staining (green: live, red: dead) for Fe-Art M+H_2O_2 after co-culturing for 30 min. After then, we change the medium and use PBS to wash the particles and observe the cells after 1 day. Experiments were repeated independently **c** per group three times with similar results. **d** The corresponding cell viability tested by CCK-8 for Fe-Art M+H_2O_2 after 30 min as shown in **a** and after 1 day as shown in **b** (n=3 independent experiments, data are presented as mean ± SD).

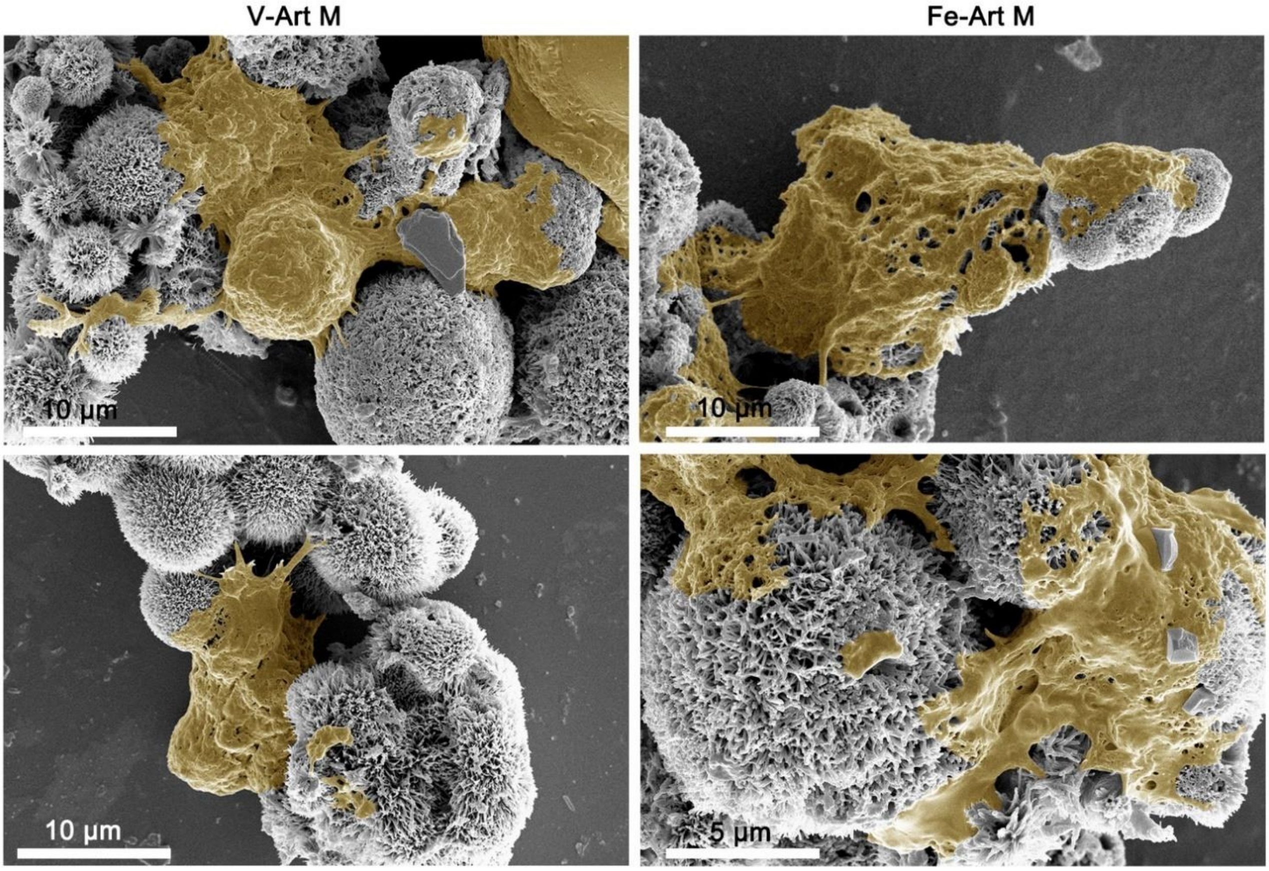

Supplementary Figure 50. SEM images of L929 cells after co-cultured with V-Art M and Fe-Art M, which indicates suspension cells adhere onto the Art Ms, but almost no cellular uptake has been observed since these hedgehog microspheres are too big for cells. Experiments were repeated independently per group three times with similar results. It is mentioned that this experiment represents the interaction of Art Ms with suspension cells.

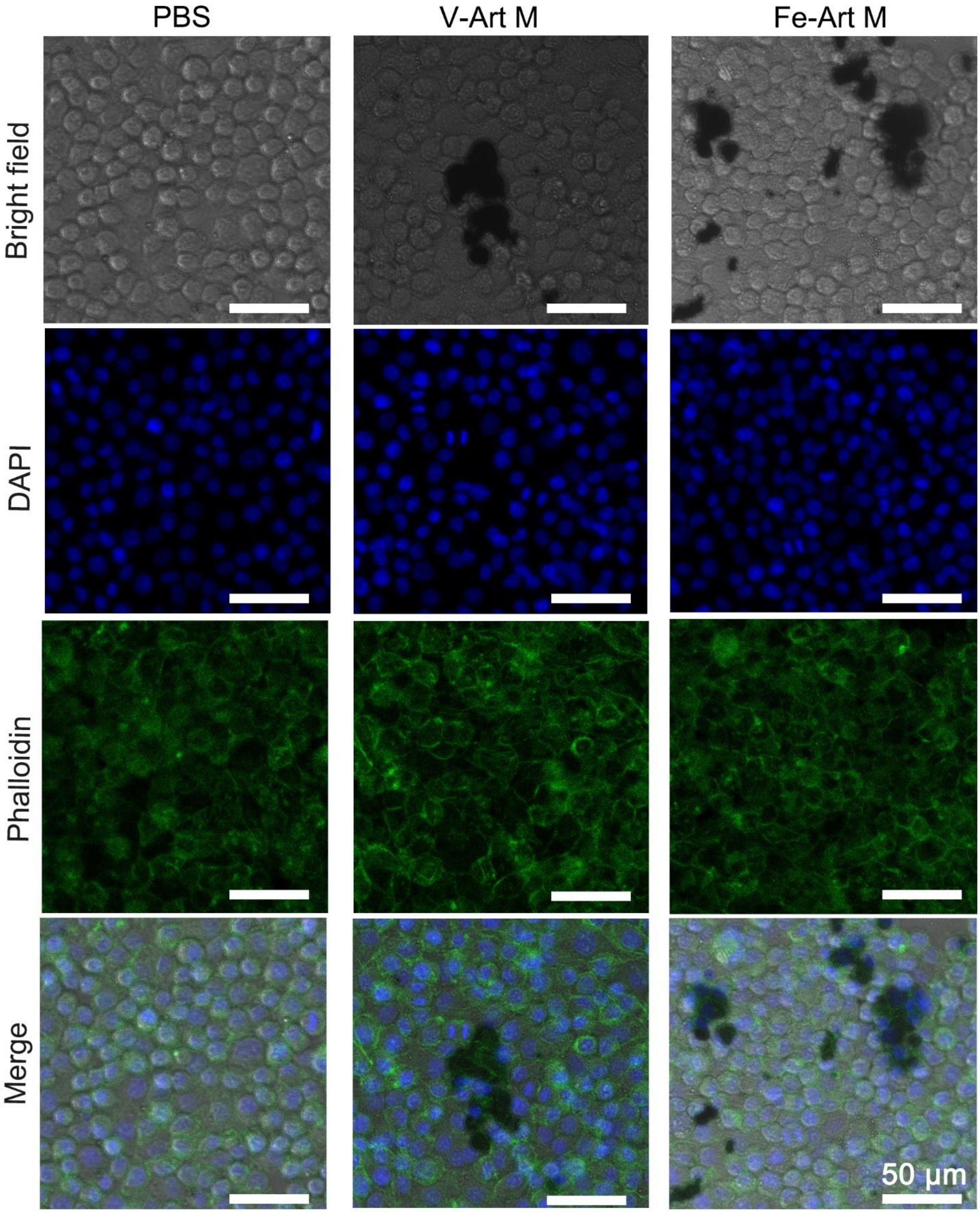

Supplementary Figure 51. Automatic inverted fluorescence microscope images of L929 cells with PBS, V-Art M, and Fe-Art M, almost no cellular apoptosis and internalization phenomenon has been observed. Experiments were repeated independently per group three times with similar results. Some of these Art Ms are even larger than the cells, which indicates that these hedgehog microspheres are difficult for cellular uptake. The co-culture time is 8 h.

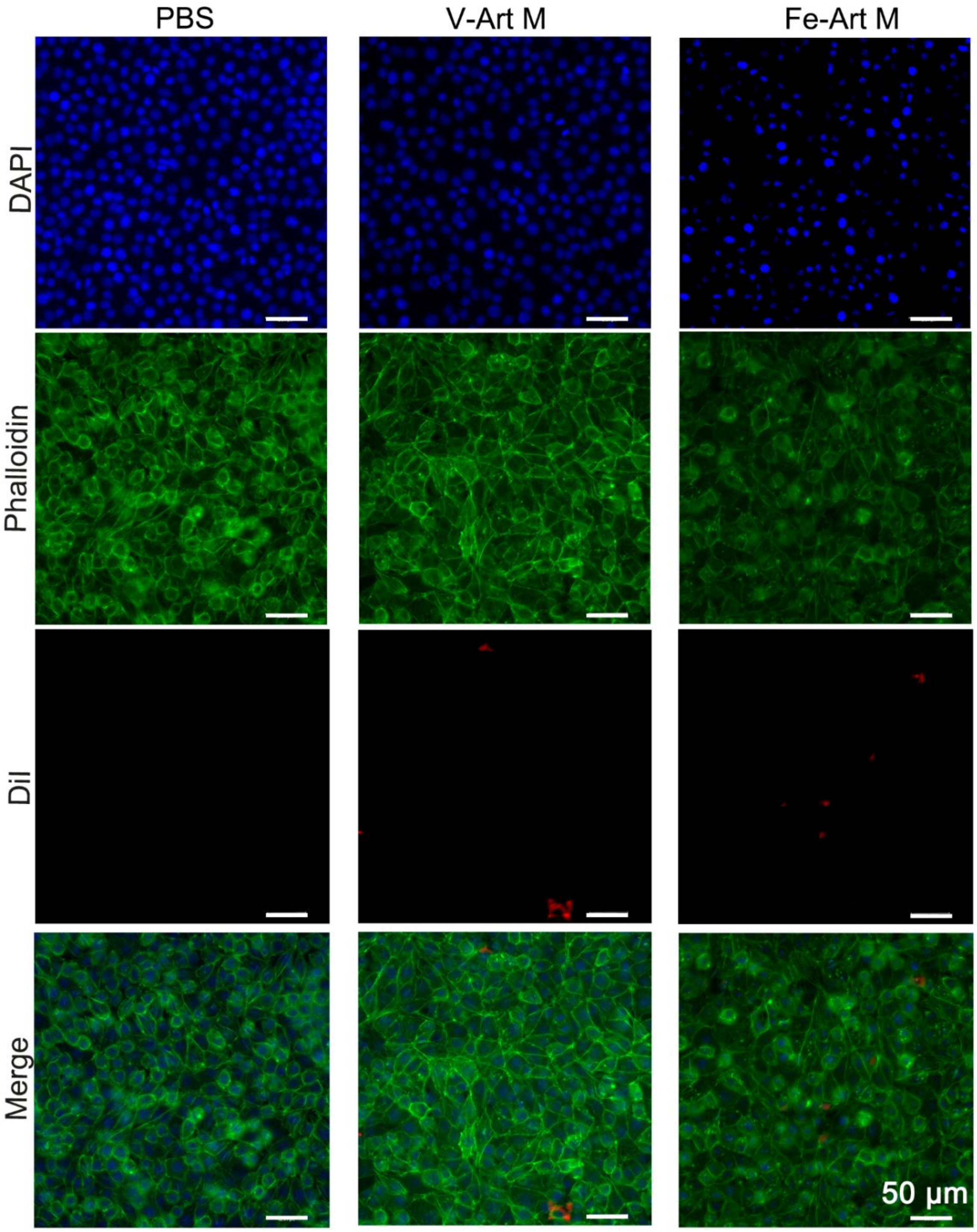

Supplementary Figure 52. The Art Ms are first labeled with DiI (dye with red fluorescence), then the Art Ms are co-cultured with L929 cells, after 8 h, the Art Ms contained media are removed to avoid the interference of these suspended Art M. Then the L929 cells are labeled with green fluorescent dye and DAPI. Experiments were repeated independently per group three times with similar results. The automatic inverted fluorescence microscope images of L929 cells with Art Ms show that there is almost no red fluorescence, which indicates that there is almost no Art Ms cellular uptake by the L929 cells.

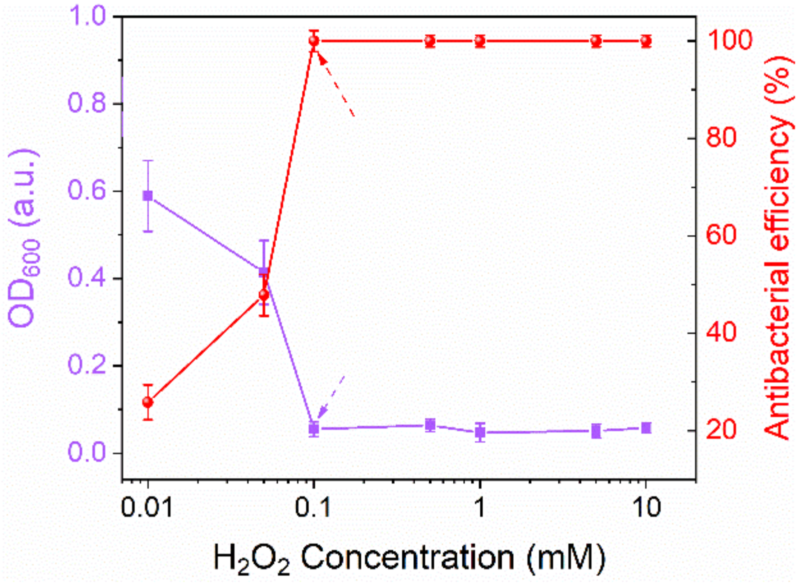

Supplementary Figure 53. The OD_{600} values and corresponding antibacterial efficiency of MRSA after being treated with Fe-Art M. The concentration of H_2O_2 is 0.01, 0.05, 0.1, 0.5, 1, 5, and 10 mM, respectively. The concentration of Fe-Art M catalyst is 10 μg/mL. When H_2O_2 concentration is 100 μM, it could realize ~100% eradication towards MRSA. Therefore, the 100 μM of H_2O_2 concentration is taken for the antibacterial studies (n=3 independent experiments per group, data are presented as mean ± SD).

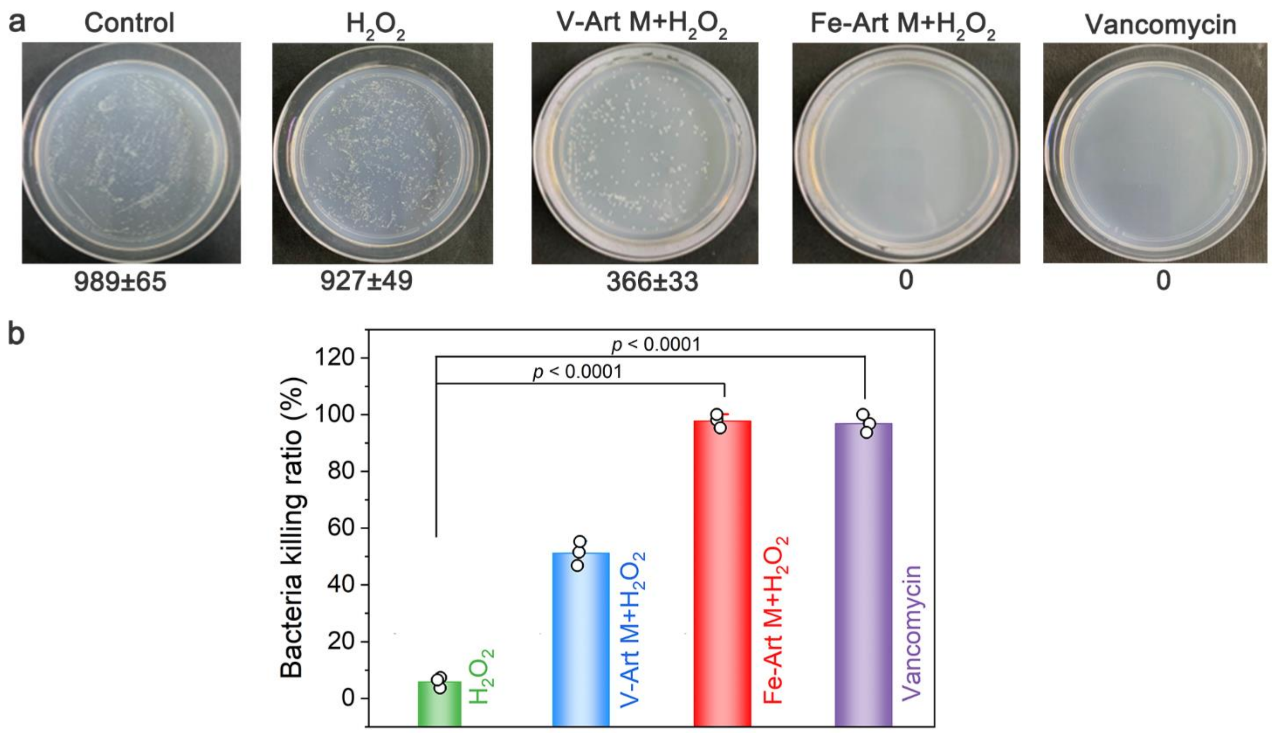

Supplementary Figure 54. a Agar plate digital photographs after treating MRSA with Art M materials. **b** MRSA killing ratio of different treatments according to numbers for the bacterial colony-forming units of MRSA estimated from panel in **a** (n=3 independent experiments per group, data are presented as mean ± SD). In **b**, *p* values are assessed by unpaired Student's two-side t tests.

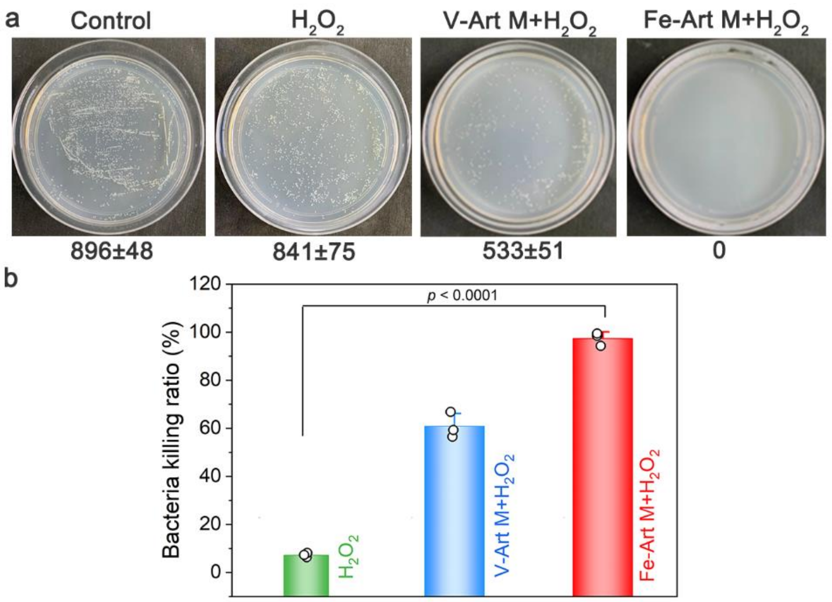

Supplementary Figure 55. a Agar plate digital photographs after treating extended-spectrum β-lactamase-producing *E. coli* with Art M materials. **b** Extended-spectrum β-lactamase-producing *E. coli* killing ratio of different treatments according to numbers for the bacterial colony-forming units estimated from panel in **a** (n=3 independent experiments per group, data are presented as mean ± SD). In **b**, *p* values are assessed by unpaired Student's two-side t tests. The almost no colony counting of Fe-Art M+ H_2O_2 group means its antibacterial effect toward Extended-spectrum β-lactamase-producing *E. coli.*

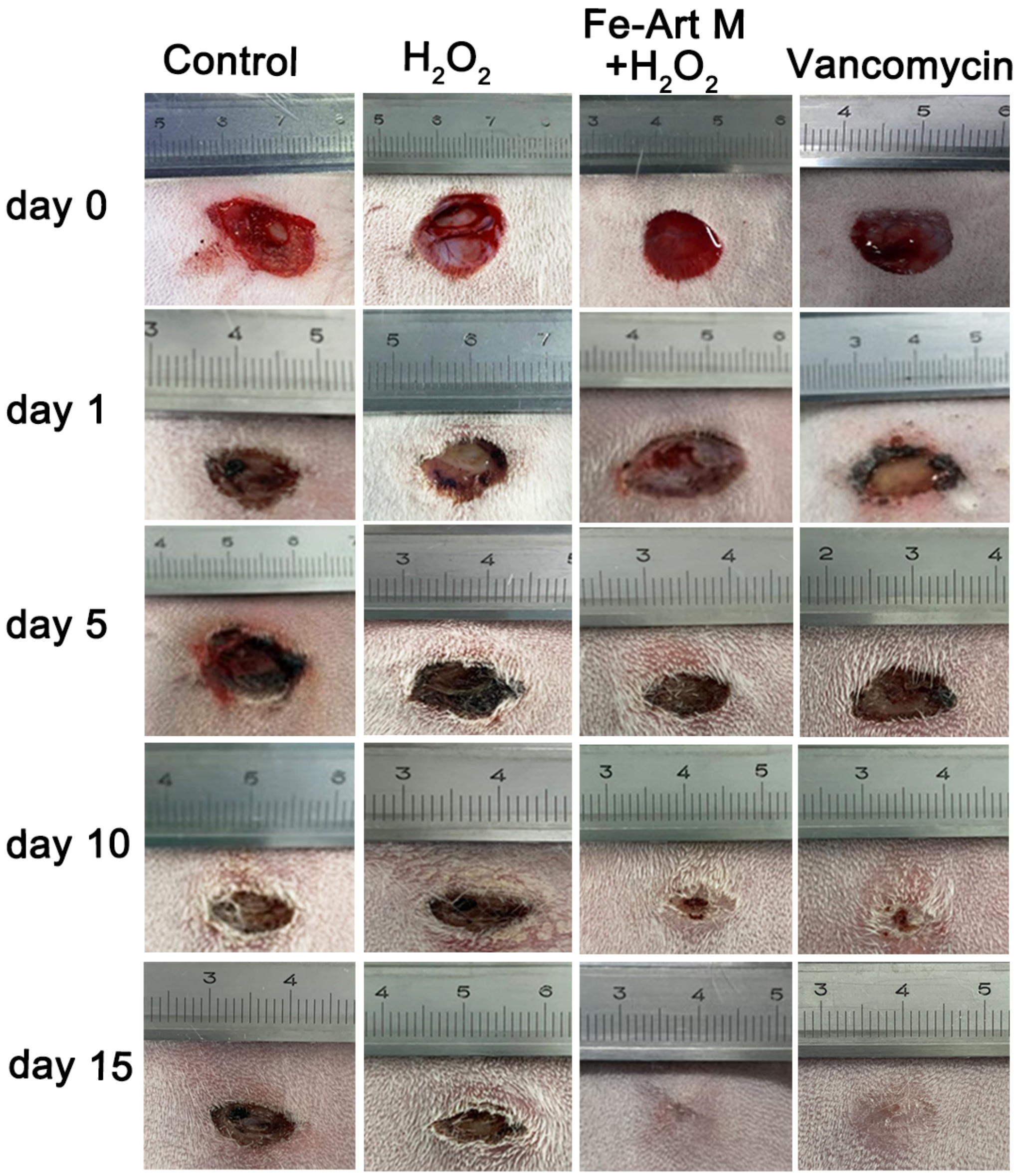

Supplementary Figure 56. Digital photographs of the wound construction, infection, disinfection, and healing.

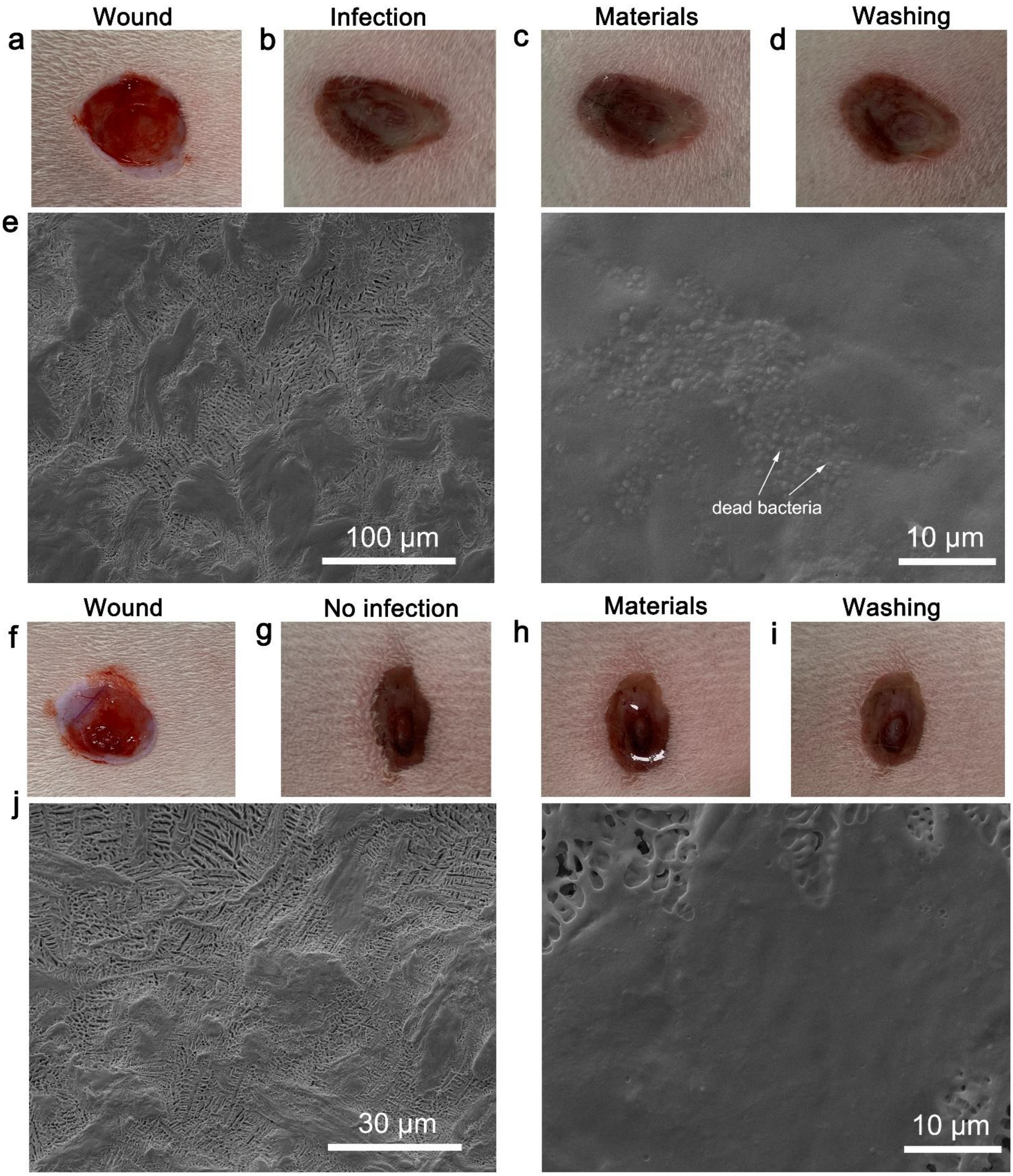

Supplementary Figure 57. The digital photographs of the **a-d** infected wound and **e** cryo-SEM images, **f-i** bare wound and **j** cryo-SEM images. It is obvious to see that there are no Fe-Art M particles on the wound, only a few of dead bacteria can be found on the wound after disinfection. Experiments were repeated independently **e**, **j** three times with similar results.

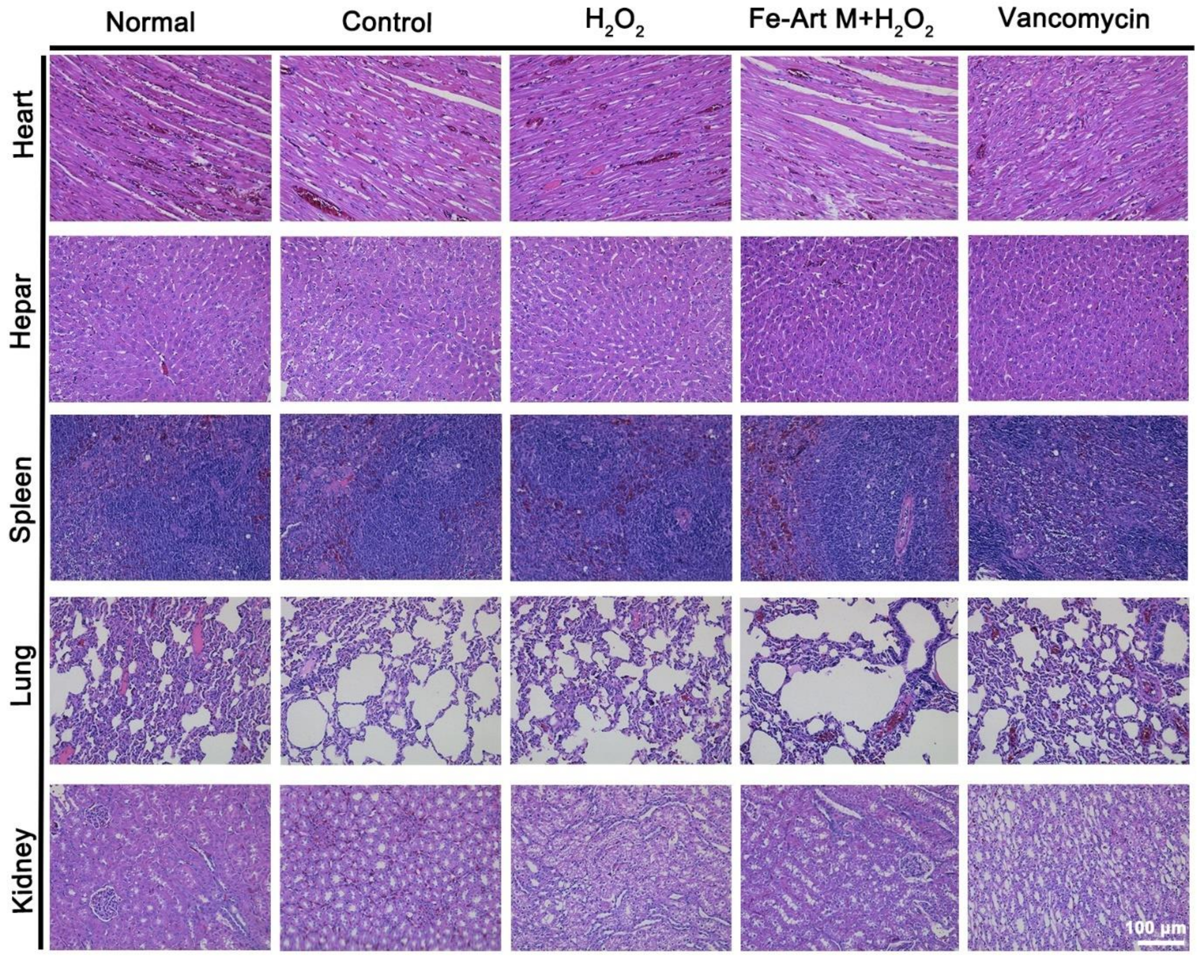

Supplementary Figure 58. HE staining images of visceral tissue slices of the rabbits with different treatments after 15 days. The healthy rabbit without MRSA infection is used as the standard group. The rabbit with MRSA infected wound is used as the control group. Experiments were repeated independently per group three times with similar results.

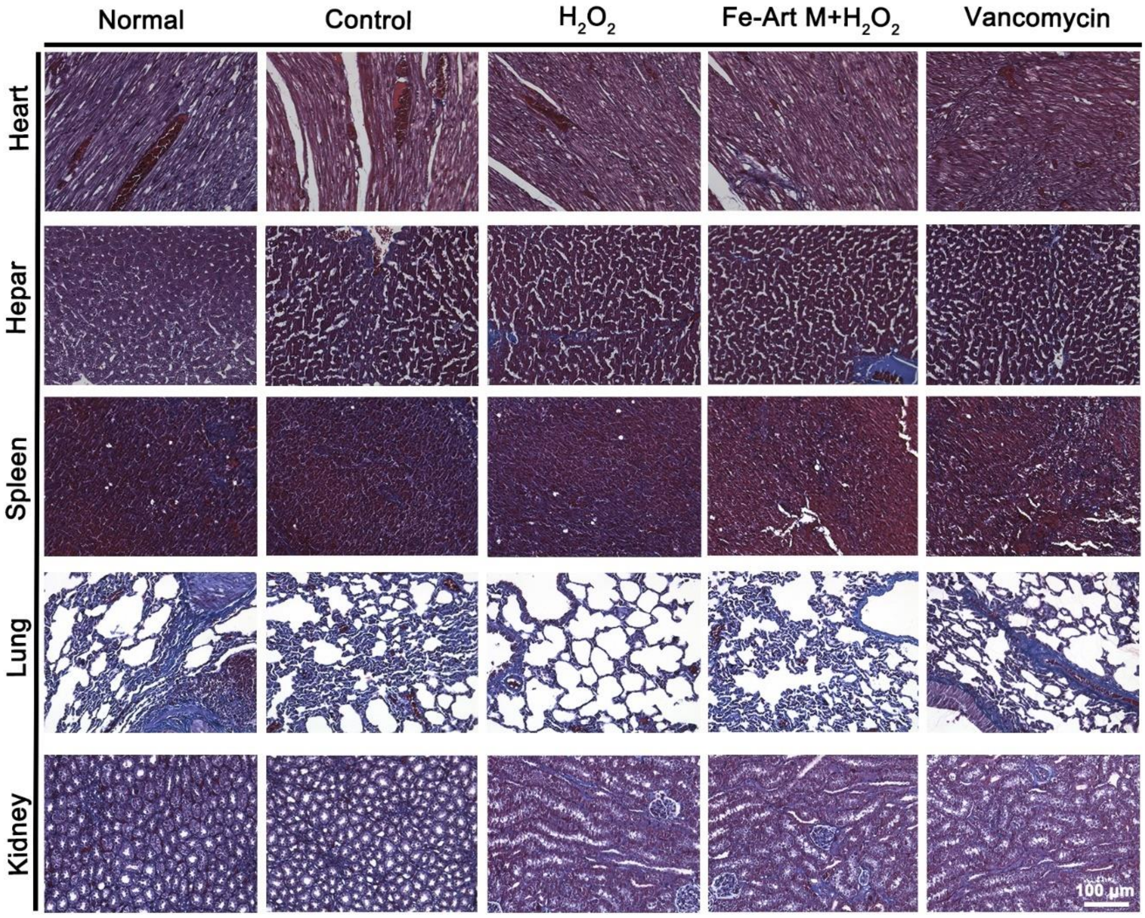

Supplementary Figure 59. Masson staining images of visceral tissue slices of the rabbits with different treatments after 15 days. The healthy rabbit without MRSA infection is used as the standard group. The rabbit with MRSA infected wound is used as the control group. Experiments were repeated independently per group three times with similar results.

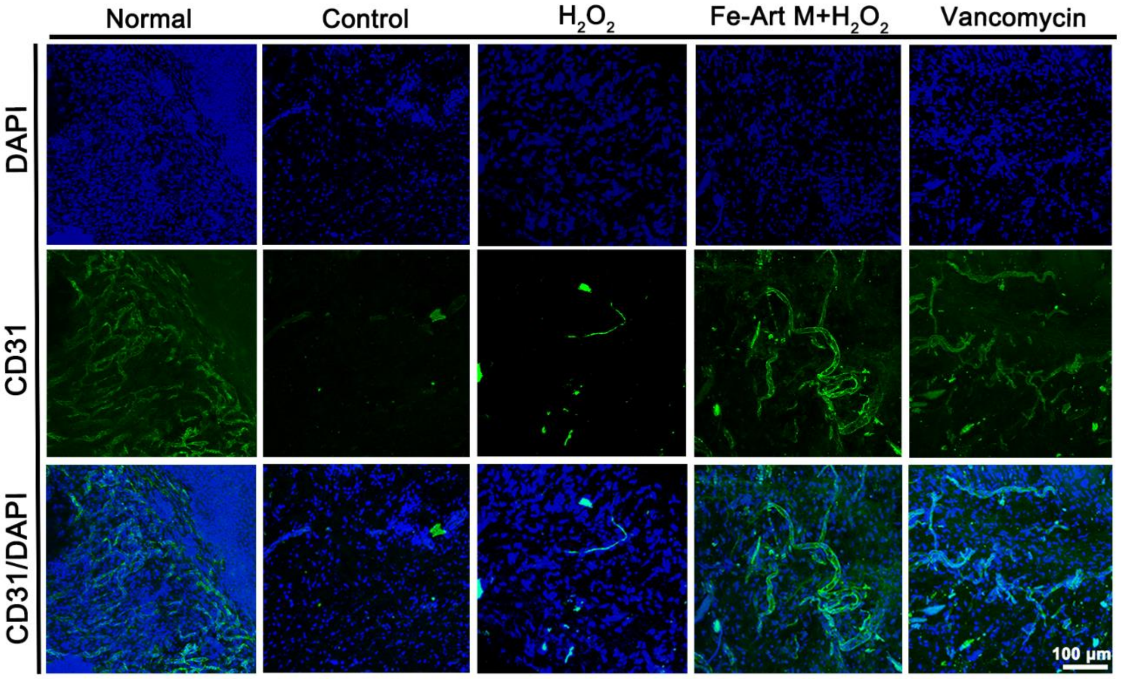

Supplementary Figure 60. Immunofluorescence staining of skin tissue for DAPI, CD31, andCD 31/DAPI. Experiments were repeated independently per group three times with similar results.

Supplementary Tables

Supplementary Table 1. The density of V/Fe-Art M and Fe-C-MPs.

Samples	V-Art M	Fe-Art M	Fe-C-MPs
ρ (g/cm^3)	0.685±0.0180	0.783±0.090	1.386±0.111

Supplementary Table 2. Elemental atomic qualification results of C, N, O, Metal (atom ratio %) in different metal-based catalysts.

Samples	C (At %)	N (At %)	O (At %)	Fe (At %)	V (At %)
Art M	96.32	1.17	2.50	0.00	0.00
V-Art M	94.41	1.2	4.28	0.00	0.11
Fe-Art M	93.80	1.64	4.45	0.11	0.00

Supplementary Table 3. Fitting results for N 1*s* spectra for diverse N species in single atoms-based catalysts.

Samples	M-N/Pyridinic N (At %) (M=metal)	Pyrrolic N (At %)	Graphitic N (At %)	Oxidized N (At %)
Art M	20.77 (398.49 eV)	4.74 (399.60 eV)	50.05 (401.10 eV)	24.44 (404.01 eV)
V-Art M	23.28 (398.70 eV)	22.12 (399.60 eV)	33.85 (401.10 eV)	20.74 (404.01 eV)
Fe-Art M	19.24 (398.89 eV)	27.17 (399.60 eV)	37.39 (401.10 eV)	16.21 (404.01 eV)

Supplementary Table 4. EXAFS fitting parameters at the Fe K-edge for various samples (S_0^2=0.729).

Sample	Shell	N^a	R(Å)^b	σ^2(Å^2)^c	ΔE_0 (eV)^d	R factor
Fe foil	Fe-Fe	8.0	2.46	0.0045	5.0	0.0017
	Fe-Fe	6.0	2.85	0.0045		
Fe_2O_3	Fe-O	6.0	2.04	0.0097	0.1	0.0009
	Fe-Fe	5.9	2.97	0.0073		
	Fe-Fe	4.2	3.39	0.0073		
	Fe-Fe	5.6	3.70	0.0073		
FePc	Fe-N	4.0	1.96	0.0036	1.2	0.0015
Fe-Art M	Fe-O/N	4.9	1.85	0.0077	-1.3	0.0008
	Fe-Fe	3.2	2.47	0.0091		
	Fe-Fe	1.8	3.18	0.0091		

N^a: coordination numbers; R^b: bond distance; σ^2: the Debye-Waller factors; ΔE_0^d: the inner potential correction. R factor: goodness of fit.

Supplementary Table 5. Comparison of the kinetic constants of V-Art M and Fe-Art M and other conventional nanozymes.

Catalysts	*K*m (mM)	V_{max} (μM s^{-1})	[E] (μM)	TON (10^{-3} s^{-1})	Ref.
V-Art M	5.130	0.051	1.882	27.300	This work
Fe-Art M	2.530	0.115	1.536	74.900	This work
MnO_2	/	0.006	114.942	0.056	[3]
Mn_2O_3	12.530	1.010	189.873	7.979	[3]
Mn_3O_4	/	0.013	131.004	0.099	[3]
CoO	92.100	1.140	133.330	8.550	[3]
CeO_2	4.410	0.180	58.139	3.096	[3]
Fe_3O_4	41.660	0.160	129.31	1.237	[3]
CuO	31.180	0.280	125.786	2.226	[3]
Co_3O_4	41.750	0.260	124.482	2.087	[3]
NiO	/	0.011	133.860	0.821	[3]
Fe-N-C	0.012	0.223	6.070	36.700	[4]
Fe-N-C SAzyme	4.310	0.620	155.490	3.990	[5]
Cu NPs/N-C	17.980	0.060	2.580	3.300	[4]
Fe-MOF	1.300	0.025	49.000	0.510	[6]
PtFe@Fe_3O_4	53.550	0.108	125.100	0.860	[7]
PtFe	217.600	0.082	0.082	2.200	[7]
Pt hollow nanodendrities (Pt HNs)	6.900	0.099	7.000	14.140	[8]

TON=V_{max}/[E], where [E] is the mole concentration of metal in the whole nanomaterials.

Supplementary Table 6. Comparison of the antibacterial efficiency and concentrations between Fe-Art M+H_2O_2 and other antibacterial agents.

Catalysts	External condition	Antibacterial action	Antibacterial concentration	Antibacterial effects	Ref.
Fe-Art M	H_2O_2	Capture and ROS generation	8 μg/mL	100% (MRSA)	This work
Vancomycin	/	/	4 μg/mL	100% (MRSA)	This work
PPPC-1	Ultrasound	ROS generation	0.04 mmol/L	100% (MRSA)	[9]
Cu_2MoS_4	Near-infrared II (NIR-II) light	NIR-II light enhanced POD/OXD	40 μg/mL	~100% (MDR *S. aureus*)	[10]
Fe-BBP	Ultrasound, H_2O_2	ROS generation	200 μg/mL	~100% (MRSA)	[11]
P5-Polymeric NPs	/	Biofilm penetration and eradication	84 μg/mL	~100% (MRSA)	[12]
HPEM	/	Membrane disruption	2.3 g/mL	99.03% (MRSA)	[13]
ANVs nanocapturer	Ultrasound	Capture and ROS generation	200 μg/mL	80% (MRSA)	[14]
Fe_3O_4/CNT/Gent	Microwave	Capture and thermal ablation	1 mg/mL	99.72% (MRSA)	[15]
PDGu(7)-b-PBLK(13)	/	Membrane disruption and interface weakening effects	256 μg/mL	99% (MRSA)	[16]
ZnPB-3	NIR	Photothermal	200 μg/mL	99.66%	[17]

		ablation and ion release		(MRSA)	
Gly-POX_{20}	/	Membrane disruption and ROS generation	25 μg/mL	100% (MRSA)	18
MoS_2/rGO VHS	Light, H_2O_2	Bacterial capture and ROS generation	50 μg/mL	100% (*S. aureus*)	19
V-POD-M	H_2O_2	Bacterial capture and ROS generation	16 μg/mL	100% (*S. aureus*)	20
PEG-MoS_2 NFs	NIR, H_2O_2	Photothermal activities and ROS generation	100 μg/mL	99% (*S. aureus*)	21
TRB-ZnO@G	NIR	Triple antibacterial activities (chemical, mechanical and photothermal)	50 μg/mL	100% (*S. aureus*)	22
C-Zn/Ag	NIR	Ions release and photothermal activities	160 μg/mL	100% (*S. aureus*)	23

PPPC: polymer-peptide-porphyrin conjugate; Fe-BBP: Fe^{3+}-BiOBr-polyethylenimine; HPEM: HCHO-PGE-MXene@PDA; ANVs: MAb-piloting nanovesicles; PDGu(7)-b-PBLK(13): poly(amido-D-glucose)-block-poly(beta-L-lysine); ZnPB-3: zinc-doped Prussian blue; Gly-POX_{20}: poly(2-oxazoline); MoS_2/rGO VHS: molybdenum disulfide/rGO vertical heterostructure; V-POD-M: virus-like peroxidase-mimic; PEG-MoS_2 NFs: polyethylene glycol functionalized molybdenum disulfide nanoflowers; TRB-ZnO@G: thermally responsive brushes-ZnO-doped carbon on graphene; C-Zn/Ag: Ag-doped carbonized ZIF nanocomposites

Supplementary Table 7. Renal function of the rabbit when treated with different conditions. (UREAL: urea (mmol/L); CREA: serum creatinine (μmol/L); BUN: blood urea nitrogen (mg/dL))

Tests	Control	H_2O_2	Vancomycin	Fe-Art M+ H_2O_2
UREAL (mmol/L)	5.51	5.35	7.23	5.25
CREA (μmol/L)	87.10	85.60	94.40	77.30
BUN (mmol/L)	5.34	5.30	6.89	5.34

Supplementary Table 8. Liver function of the rabbit when treated with different conditions. (ALT: alanine aminotransferase (U/L); AST: aspartate aminotransferase (U/L); TP: total protein (g/L); ALB: albumin (g/L); GLB: globulin (g/L); A/G: albumin globulin ratio)

Tests	Control	H_2O_2	Vancomycin	Fe-Art M+ H_2O_2
ALT	50.00	57.00	56.00	57.00
AST	18.90	31.00	37.50	31.70
TP	62.00	63.30	51.10	65.50
ALB	55.60	54.0	48.30	54.60
GLB	6.40	9.30	2.80	10.90
A/G	8.69	5.81	17.25	5.01

Supplementary Methods

Synthesis of Fe-C-MPs with a smooth surface: The synthetic process of Fe-doped carbonous micro-particles (Fe-C-MPs) was divided into two steps. Firstly, the micrometer-scale zeolitic imidazolate framework-8 (ZIF-8) large crystals were synthesized by a hydrothermal transformation method.[24] 588 mg (1.977 mmol) $Zn(NO_3)_2{\cdot}6H_2O$ was dissolved in 40 mL methanol. 324 mg (3.954 mmol) 2-methylimidazole and 538 mg (7.908 mmol) sodium formate were dissolved in 40 mL methanol. Then, the latter solution was poured into the former solution under continuous stirring. The solution was heated at 90 °C for 24 hours in a sealed glass jar. The crystals were recovered by centrifuged, washed with methanol, and dried under vacuum. Afterward, with a heating ramp rate of 5 °C/min, the as-prepared ZIF-8 large crystals were transferred into a ceramic boat, placed in a tube furnace, and heated to 1100 °C for 1 h to obtain carbonized micro-particles (C-ZIF-8). After chilling down to room temperature, the products was gathered and washed with HCl (1 M) solution at 80 °C for further use. Subsequently, 0.02 g of the micro-particles was dispersed in 2.5 mL of ethanol solution, and then a 30 μL mixture (metal acetylacetonate and 1,10-phenanthroline with a molar ratio of 2:1) was added. Finally, the dispersion was dried in a vacuum oven overnight and heated to 600 °C in an N_2 atmosphere for 2 h to obtain Fe-C-MPs with a smooth surface. This Fe doped micro-particle with a smooth surface was used as the control group to our hedgehog micro-particles for antibacterial studies.

Analysis of free radicals by fluorescent agents:

•O_2^- probe analysis: HE (hydroethidine) was pre-dissolved in DMSO (1 mg/mL) as an indicator for •O_2^-. 100 μL of catalysts (0.1 mg/mL) were treated with 5 μL H_2O_2 (1 M). After reaction for 5 min, 400 μL of HE was added. The excitation wavelength is 470 nm.

$^1O_2$ probe analysis: 100 μL of catalysts (0.1 mg/mL) were treated with 5 μL H_2O_2 (1 M); after reaction for 5 min, 10 μL of SOSG (singlet oxygen sensor green) and 400 μL of buffer was added. The excitation wavelength is 490 nm.

HClO probe analysis: 100 μL of catalysts (0.1 mg/mL) were treated with 5 μL H_2O_2 (1 M), after reaction for 5 min, 10 μL of APF (aminophenyl fluorescein) and 400 μL of buffer was added. The excitation wavelength is 490 nm.

Cell Interaction Experiments: The interaction between Art M and L929 cells was visualized via SEM. After incubation of suspended cells with V/Fe-Art M for about 1 h, the cell suspensions were fixed with 2.5 wt.% glutaraldehyde, dehydrated with a gradient of ethanol/water solution, and treated with supercritical drying. Then, the SEM images were obtained to observe their interactions and morphologies.

The L929 cells with V/Fe-Art M were further investigated by fluorescence microscopy to observe their interaction and cellular morphology. For fluorescence microscopy observation, L929 cells were seeded (3×10^5 cells in 1 mL DMEM) into the 24-well plate and allowed to adhere overnight. Then the culture media were replaced by V/Fe-Art M (1 mL, 0.02 mg/mL) in DMEM. After varied co-incubation duration (8 h), DAPI/Phalloidin was applied to stain cell nuclei and cytoskeleton for 5 h.

Supplementary References:

1 Zhou, G. *et al.* Theoretical Calculation Guided Design of Single-Atom Catalysts toward Fast Kinetic and Long-Life Li–S Batteries. *Nano Lett.* **20**, 1252-1261 (2020).

2 Natalio, F. *et al.* Vanadium pentoxide nanoparticles mimic vanadium haloperoxidases and thwart biofilm formation. *Nat. Nanotechnol.* **7**, 530-535 (2012).

3 Wang, X. *et al.* e_g occupancy as an effective descriptor for the catalytic activity of perovskite oxide-based peroxidase mimics. *Nat. Commun.* **10**, 704 (2019).

4 Wu, Y. *et al.* Cascade Reaction System Integrating Single-Atom Nanozymes with Abundant Cu Sites for Enhanced Biosensing. *Anal. Chem.* **92**, 3373-3379 (2020).

5 Jiao, L. *et al.* Densely Isolated FeN_4 Sites for Peroxidase Mimicking. *ACS Catal.* **10**, 6422-6429 (2020).

6 Xu, W. *et al.* Glucose Oxidase-Integrated Metal–Organic Framework Hybrids as Biomimetic Cascade Nanozymes for Ultrasensitive Glucose Biosensing. *ACS Appl. Mater. Interfaces* **11**, 22096-22101 (2019).

7 Li, S. *et al.* A Nanozyme with Photo-Enhanced Dual Enzyme-Like Activities for Deep Pancreatic Cancer Therapy. *Angew. Chem. Int. Ed.* **58**, 12624-12631 (2019).

8 Ge, C. *et al.* Synthesis of Pt Hollow Nanodendrites with Enhanced Peroxidase-Like Activity against Bacterial Infections: Implication for Wound Healing. *Adv. Funct. Mater.* **28**, 1801484 (2018).

9 Wang, D. *et al.* Precise magnetic resonance imaging-guided sonodynamic therapy for drug-resistant bacterial deep infection. *Biomaterials* **264**, 120386 (2021).

10 Shan, J. *et al.* Cu_2MoS_4 Nanozyme with NIR-II Light Enhanced Catalytic Activity for Efficient Eradication of Multidrug-Resistant Bacteria. *Small* **16**, 2001099 (2020).

11 Song, M. *et al.* Sonoactivated Chemodynamic Therapy: A Robust ROS Generation Nanotheranostic Eradicates Multidrug-Resistant Bacterial Infection. *Adv. Funct. Mater.* **30**, 2003587 (2020).

12 Gupta, A. *et al.* Engineered Polymer Nanoparticles with Unprecedented Antimicrobial Efficacy and Therapeutic Indices against Multidrug-Resistant Bacteria and Biofilms. *J. Am. Chem. Soc.* **140**, 12137-12143 (2018).

13 Zhou, L. *et al.* Conductive Antibacterial Hemostatic Multifunctional Scaffolds Based on $Ti_3C_2T_x$ MXene Nanosheets for Promoting Multidrug-Resistant Bacteria-Infected Wound Healing. *ACS Nano* **15**, 2468-2480 (2021).

14 Pang, X. *et al.* Sono-Immunotherapeutic Nanocapturer to Combat Multidrug-Resistant Bacterial Infections. *Adv. Mater.* **31**, 1902530 (2019).

15 Qiao, Y. *et al.* Treatment of MRSA-infected osteomyelitis using bacterial capturing, magnetically targeted composites with microwave-assisted bacterial killing. *Nat. Commun.* **11**, 4446 (2020).

16 Zhang, K. *et al.* Enantiomeric glycosylated cationic block co-beta-peptides eradicate *Staphylococcus aureus* biofilms and antibiotic-tolerant persisters. *Nat. Commun.* **10**, 4792 (2019).

17 Li, J. *et al.* Zinc-doped Prussian blue enhances photothermal clearance of *Staphylococcus aureus* and promotes tissue repair in infected wounds. *Nat. Commun.* **10**, 4490 (2019).

18 Zhou, M. *et al.* Poly(2-Oxazoline)-Based Functional Peptide Mimics: Eradicating MRSA Infections and Persisters while Alleviating Antimicrobial Resistance. *Angew. Chem. Int. Ed.* **59**,

6412-6419 (2020).
19 Wang, L. *et al.* Defect-Rich Adhesive Molybdenum Disulfide/rGO Vertical Heterostructures with Enhanced Nanozyme Activity for Smart Bacterial Killing Application. *Adv. Mater.* **32**, 2005423 (2020).
20 Yang, Y. *et al.* Bioinspired Spiky Peroxidase-Mimics for Localized Bacterial Capture and Synergistic Catalytic Sterilization. *Adv. Mater.* **33**, 2005477 (2021).
21 Yin, W. *et al.* Functionalized Nano-MoS_2 with Peroxidase Catalytic and Near-Infrared Photothermal Activities for Safe and Synergetic Wound Antibacterial Applications. *ACS Nano* **10**, 11000-11011 (2016).
22 Fan, X. *et al.* Metal–Organic-Framework-Derived 2D Carbon Nanosheets for Localized Multiple Bacterial Eradication and Augmented Anti-infective Therapy. *Nano Lett.* **19**, 5885-5896 (2019).
23 Yang, Y. *et al.* Metal–Organic Framework/Ag-Based Hybrid Nanoagents for Rapid and Synergistic Bacterial Eradication. *ACS Appl. Mater. Interfaces* **12**, 13698-13708 (2020).
24 Zhang, C. *et al.* Unexpected Molecular Sieving Properties of Zeolitic Imidazolate Framework-8. *J. Phys. Chem. Lett.* **3**, 2130-2134 (2012).
