# Supplementary material for: Hedgehog artificial macrophage with atomic-catalytic centers to combat Drug-resistant bacteria
Source: Nat Commun. 2021 Oct 22;12:6143. doi: 10.1038/s41467-021-26456-9 (PMC8536674; doi:10.1038/s41467-021-26456-9)
Supplement: Supplementary file 3 — Reporting Summary [file 41467_2021_26456_MOESM3_ESM.pdf]

nature research

Corresponding author(s): Chong Cheng

Last updated by author(s): Apirl 12, 2021

Reporting Summary

Nature Research wishes to improve the reproducibility of the work that we publish. This form provides structure for consistency and transparency in reporting. For further information on Nature Research policies, see our Editorial Policies and the Editorial Policy Checklist.

Statistics

For all statistical analyses, confirm that the following items are present in the figure legend, table legend, main text, or Methods section.

n/a	Confirmed	
☐	☒	The exact sample size (n) for each experimental group/condition, given as a discrete number and unit of measurement
☐	☒	A statement on whether measurements were taken from distinct samples or whether the same sample was measured repeatedly
☐	☒	The statistical test(s) used AND whether they are one- or two-sided<br>*Only common tests should be described solely by name; describe more complex techniques in the Methods section.*
☒	☐	A description of all covariates tested
☐	☒	A description of any assumptions or corrections, such as tests of normality and adjustment for multiple comparisons
☐	☒	A full description of the statistical parameters including central tendency (e.g. means) or other basic estimates (e.g. regression coefficient) AND variation (e.g. standard deviation) or associated estimates of uncertainty (e.g. confidence intervals)
☐	☒	For null hypothesis testing, the test statistic (e.g. F, t, r) with confidence intervals, effect sizes, degrees of freedom and P value noted<br>*Give P values as exact values whenever suitable.*
☒	☐	For Bayesian analysis, information on the choice of priors and Markov chain Monte Carlo settings
☒	☐	For hierarchical and complex designs, identification of the appropriate level for tests and full reporting of outcomes
☒	☐	Estimates of effect sizes (e.g. Cohen's d, Pearson's r), indicating how they were calculated

Our web collection on statistics for biologists contains articles on many of the points above.

Software and code

Policy information about availability of computer code

Data collection	Confocal laser scanning microscope (CLSM, N-STORM & A1, Nikon) and fluorescence microscopy (DMIRE2, Leica) were used for cellular imaging and data collection
Data analysis	Origin 9.0, VASP 5.4.1, GraphPad Prism 7.0, MDI Jade 6, Avantage 5.967, Athena software 0.9.26, GMS-freeanalysis 4, and NIS-Elements Viewer 5.21 were used to analyze the data.

For manuscripts utilizing custom algorithms or software that are central to the research but not yet described in published literature, software must be made available to editors and reviewers. We strongly encourage code deposition in a community repository (e.g. GitHub). See the Nature Research guidelines for submitting code & software for further information.

Data

Policy information about availability of data

All manuscripts must include a data availability statement. This statement should provide the following information, where applicable:

- Accession codes, unique identifiers, or web links for publicly available datasets
- A list of figures that have associated raw data
- A description of any restrictions on data availability

All data are available in the main text or the Supplementary Information. Source data are provided with this paper.

Field-specific reporting

Please select the one below that is the best fit for your research. If you are not sure, read the appropriate sections before making your selection.

- [x] Life sciences
- [ ] Behavioural & social sciences
- [ ] Ecological, evolutionary & environmental sciences

For a reference copy of the document with all sections, see nature.com/documents/nr-reporting-summary-flat.pdf

Life sciences study design

All studies must disclose on these points even when the disclosure is negative.

Sample size	All biologically based assays were performed with the usual and sufficient sample size setting according to earlier paper (Nat. Commun. 2021, 12, 114). These sample sizes were sufficient for a statistical analysis. All experiments reported here have n number and repetitions reported.
Data exclusions	No data was excluded from the analysis.
Replication	Results shown in the manuscript are representative of at least three independent experiments. All our attempts at replication were successful with similar results.
Randomization	Our samples/organisms were allocated randomly.
Blinding	In all experiments, investigators were blinded to group allocation during data collection and processing.

Reporting for specific materials, systems and methods

We require information from authors about some types of materials, experimental systems and methods used in many studies. Here, indicate whether each material, system or method listed is relevant to your study. If you are not sure if a list item applies to your research, read the appropriate section before selecting a response.

Materials & experimental systems

n/a	Involved in the study	
x		Antibodies
	x	Eukaryotic cell lines
x		Palaeontology and archaeology
	x	Animals and other organisms
x		Human research participants
x		Clinical data
	x	Dual use research of concern

Methods

n/a	Involved in the study	
x		ChIP-seq
x		Flow cytometry
x		MRI-based neuroimaging

Eukaryotic cell lines

Policy information about cell lines

Cell line source(s)	Mouse fibroblast (L929), commercial source is ATCC company (CCL-1)
Authentication	The cell line was not authenticated.
Mycoplasma contamination	Cell line were not contaminated by Mycoplasma.
Commonly misidentified lines (See ICLAC register)	No commonly misidentified cell line was used.

Animals and other organisms

Policy information about studies involving animals; ARRIVE guidelines recommended for reporting animal research

Laboratory animals	New Zealand White Rabbit, male, 8-12 weeks.
Wild animals	The study did not involve wild animals.
Field-collected samples	No field-collected samples were used in this study.
Ethics oversight	The ethical oversight is subject to the approval and guidance of the animal ethical standard from Animal Ethics Committee in West China Hospital, Sichuan University, Chengdu, China.

Note that full information on the approval of the study protocol must also be provided in the manuscript.

Dual use research of concern

Policy information about dual use research of concern

Hazards

Could the accidental, deliberate or reckless misuse of agents or technologies generated in the work, or the application of information presented in the manuscript, pose a threat to:

No	Yes	
☒	☐	Public health
☒	☐	National security
☒	☐	Crops and/or livestock
☒	☐	Ecosystems
☒	☐	Any other significant area

Experiments of concern

Does the work involve any of these experiments of concern:

No	Yes	
☒	☐	Demonstrate how to render a vaccine ineffective
☒	☐	Confer resistance to therapeutically useful antibiotics or antiviral agents
☒	☐	Enhance the virulence of a pathogen or render a nonpathogen virulent
☒	☐	Increase transmissibility of a pathogen
☒	☐	Alter the host range of a pathogen
☒	☐	Enable evasion of diagnostic/detection modalities
☒	☐	Enable the weaponization of a biological agent or toxin
☒	☐	Any other potentially harmful combination of experiments and agents
